# Computer Simulations Show That Liquid–Liquid Phase Separation Enhances Self-Assembly

**DOI:** 10.1021/acsnano.5c08120

**Published:** 2025-08-09

**Authors:** Layne B. Frechette, Naren Sundararajan, Fernando Caballero, Anthony Trubiano, Michael F. Hagan

**Affiliations:** Martin Fisher School of Physics, Brandeis University, Waltham, Massachusetts 02453, United States

**Keywords:** virus capsids, self-assembly, liquid−liquid phase separation, biomolecular condensates, molecular dynamics, computer simulations

## Abstract

Biomolecular condensates are liquid- or gel-like droplets of proteins and nucleic acids formed at least in part through liquid–liquid phase separation. Condensates enable diverse functions of cells and the pathogens that infect them, including self-assembly reactions. For example, it has been shown that many viruses form condensates within their host cells to compartmentalize capsid assembly and packaging of the viral genome. Yet, the physical principles controlling condensate-mediated self-assembly remain incompletely understood. In this article, we use coarse-grained molecular dynamics simulations to study the effect of a condensate on the assembly of icosahedral capsids. The capsid subunits are represented by simple shape-based models to enable simulating a wide range of length and time scales, while the condensate is modeled implicitly to study the effects of phase separation independent of the molecular details of biomolecular condensates. Our results show that condensates can significantly enhance assembly rates, yields, and robustness to parameter variations, consistent with previous theoretical predictions. However, extending beyond those predictions, the computational models also show that excluded volume enables control over the number of capsids that assemble within condensates. Moreover, long-lived aberrant off-pathway assembly intermediates can suppress yields within condensates. In addition to elucidating condensate-mediated assembly of viruses and other biological structures, these results may guide the use of condensates as a generic route to enhance and control self-assembly in human-engineered systems.

## Graphical abstract

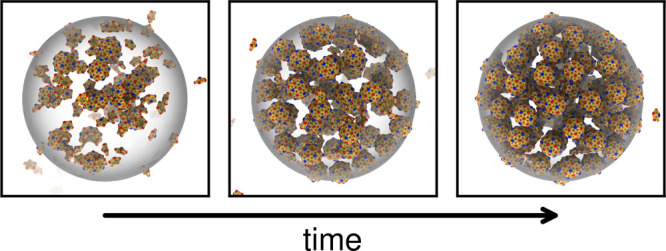

## Introduction

Many critical biological functions rely on high-fidelity assembly of structures, such as viral capsids,^1–8^ photonic nanostructures,^9^ bacterial microcompartments,^10–17^ and other proteinaceous organelles.^18–22^ These structures form with astonishing robustness, despite the complex and highly adaptive nature of the cellular cytoplasm. Self-assembly also has promising technological applications, such as enabling highly scalable bottom-up manufacturing of nanostructured materials.^23–25^ Recent developments in DNA origami, protein design, supramolecular assembly, and patchy-colloidal particles have enabled the design of human-engineered subunits that assemble into complex architectures including icosahedral shells and cylindrical tubules with programmable sizes (e.g.,^26–67^ ). However, in contrast to biology, the ability to achieve high-fidelity assembly in these synthetic systems is far from robust, requiring precise tuning of subunit concentrations and interactions to avoid kinetic traps (long-lived off-pathway intermediates).^8,31,68–87^ Biological systems employ multiple mechanisms to avoid such kinetic traps. In this article we use computer simulations to investigate one of these mechanisms—self-assembly coupled to biomolecular condensates. Our results show that condensates can significantly increase assembly rates and robustness, and enable controlling the final yield.

It has become clear that biomolecular condensates formed through liquid–liquid phase separation (LLPS) play a key role in spatially organizing cellular environments.^88–92^ These ‘membraneless organelles’ are involved in essential processes, such as transcriptional regulation,^93–97^ cell division,^98,99^ and neuronal synapse formation.^100–102^ More recent evidence suggests that condensates can also control self-assembly within cells. Examples include the assembly of clathrin cages during endocytosis,^103^ the formation of postsynaptic densities^104^ and presynaptic vesicle release sites^105,106^ at neuronal synapses, aggregation of α-synuclein,^107^ and actin assembly in polypeptide coacervates.^108^ In addition to normal biological functions, condensates are also critical for pathogenic infections. For example, in many viral infections, the outer protein shells (capsids) assemble within condensates known as virus factories, replication sites, Negri bodies, inclusion bodies, or viroplasms.^109–128^ However, the effects of LLPS on assembly and the benefits to the viral lifecycle remain incompletely understood.

Extensive theoretical and computational works have investigated how condensates form and how they are controlled by chemical reactions and other nonequilibrium processes (e.g.,^95,96,129–166^ ). However, there have been comparatively few studies of condensate-coupled assembly. Recent investigations using chemical kinetics-based rate equations suggest that preferential partitioning of subunits into condensates can significantly enhance assembly rates by locally concentrating the subunits.^107,133,167–170^ Ref 170 also showed that LLPS can make assembly more robust to parameter variations, significantly broadening the range of subunit concentrations or binding affinities that lead to productive assembly. Although Bartolucci et al.^169^ made a comprehensive study that included effects of subunits on condensate phase coexistence, in many systems the subunits remain sufficiently dilute within condensates so that their effects on condensate stability can be neglected (i.e., subunits are “clients” rather than “scaffolds”^171^). Despite the important insights from these works, they were limited to rate equation models that assume spatially uniform concentrations within condensates. Further, they must preassume the set of allowed assembly intermediates and thus have not considered the excluded volume geometries of assembly structures or the possibility of malformed structures and other off-pathway intermediates that can limit assembly of target structures (e.g.,^3,5,8,28,69–73,78–84,172–186^ ).

In this article, we use molecular dynamics simulations to avoid these approximations. To enable conclusions that are independent of the molecular details of condensates and to simulate a wide range of lengths and time scales, we implicitly model the condensate. That is, we represent the condensate as a spherical region with an attractive field that drives partitioning of subunits into the condensate. The simulation results are consistent with many of the predictions of the rate equation models, such as enhanced yields, rates and robustness to parameter variations, but also identify behaviors that are not captured in rate equation models. For example, capsid yields are suppressed by kinetic traps due to aberrant off-pathway structures for strong subunit binding affinities. Capsid yields also drop precipitously when the concentration of subunits approaches the close-packing limit. We develop an equilibrium theory that recapitulates this behavior, indicating that this reduction in yield is at least partially thermodynamic in origin. In this limit, we observe arrays of nearly closely packed capsids, which are highly reminiscent of recent electron microscopy images of virus factories (Figure 1). We show that capsid yields can be precisely controlled by the condensate volume. In addition to shedding light on viral lifecycles, this suggests a strategy to achieve size-controlled crystalline arrays in synthetic assembly systems.

**Figure 1:**
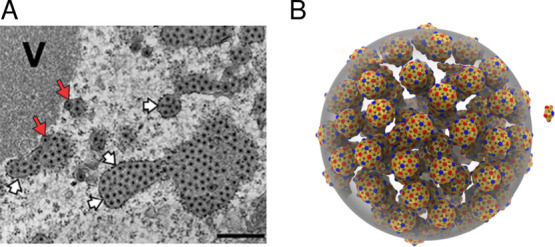
Densely packed viral factories in experiments and computer simulations. (A) Electron microscopy image from a rotavirus-infected cell. Gray regions are viroplasms, and black dots are assembled capsids. V, viroplasm; long red arrows, enveloped viral particles; short white arrows, endoplasmic reticulum surrounding viroplasms. Reprinted (Adapted or Reprinted in part) with permission under a Creative Commons CC BY 4.0 License from Papa, G. et al. Recombinant Rotaviruses Rescued by Reverse Genetics Reveal the Role of NSP5 Hyperphosphorylation in the Assembly of Viral Factories. Journal of Virology 2019, 94, e01110-19. Copyright 2019 Papa et al. (B) Snapshot from a computer simulation of LLPS-coupled capsid assembly, showing capsids (small spherical particles) densely packed within a condensate (large gray sphere).

## Results

We use molecular dynamics to simulate *N* subunits in a fixed volume *V* at a temperature *T*. We study assembly across a range of total subunit concentrations ρ_T_ = *N*/*V*. To assess the generality of our results, we study two capsid models, which differ in complexity and in the size of the assembled structures. In the first model (Figure 2A–C), inspired by SV40 virus capsids,^187,188^ pentagonal subunits (Figure 2A) assemble into dodecahedral capsids (Figure 2C). The second, more complex, model (Figure 2D-F) is inspired by DNA origami assembly, and consists of triangular subunits (Figure 2D) that assemble into *T* = 1 icosahedral capsids (Figure 2F).^31^ See Subunit models for additional model details.

**Figure 2:**
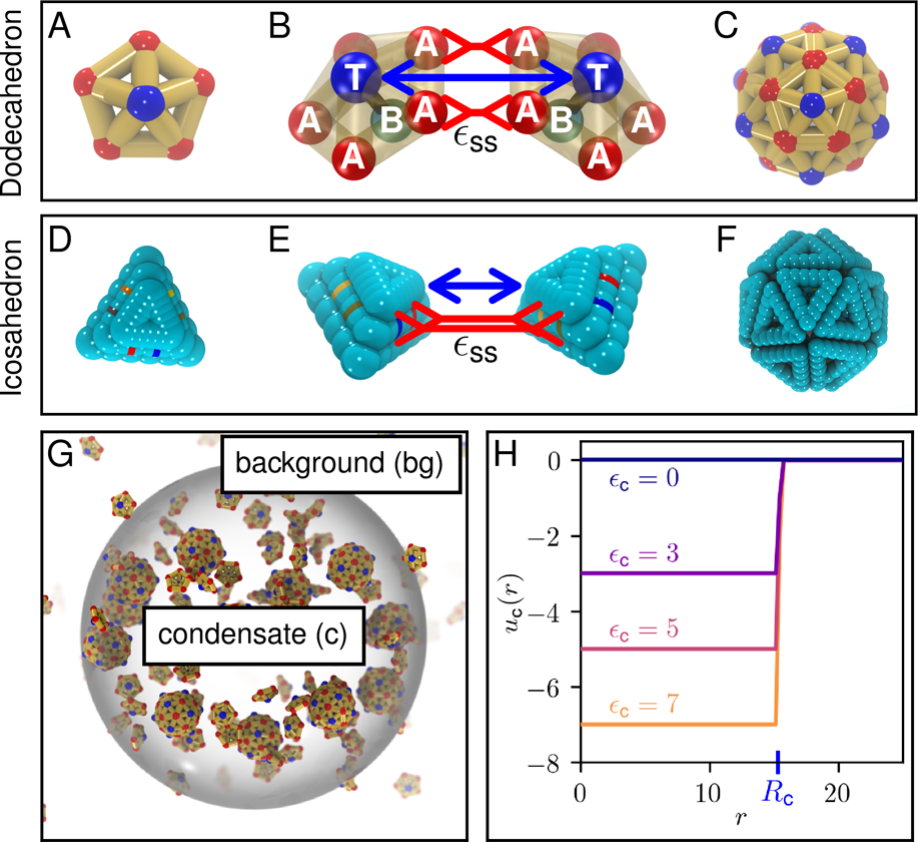
Illustration of the capsid models. (A) Dodecahedron model subunit. (B) Interactions between dodecahedron subunits. Top (‘T’) pseudoatoms repel each other, while attractor (‘A’) pseudoatoms attract each other with interaction strength ϵ_ss_. Bottom (‘B’) pseudoatoms repel ‘T’ pseudoatoms, helping to prevent misbinding. (C) Dodecahedral capsid. (D) Icosahedron model subunit. (E) Interactions between icosahedron subunits. Excluder pseudoatoms (cyan) repel each other, while attractor pseudoatoms (all other colors) attract each other with interaction strength ϵ_ss_. (F) Icosahedral capsid. (G) Snapshot of a condensate containing monomeric subunits and capsids, taken from an assembly simulation of the dodecahedron model. Subunits can exchange between the condensate (gray sphere) with volume *V*_c_ and the background with volume *V*_bg_. At equilibrium, the concentrations of monomers in the condensate and background are related by the partition coefficient, *K*_c_ = ρ_1_^c^/ρ_1_^bg^. (H) Plot of the condensate potential *u*_c_(*r*) (eq 7) as a function of distance *r* from the center of the condensate for indicated values of the well depth ϵ_c_. The value *R*_c_, marked in blue on the *x* axis, denotes the condensate radius (here *R*_c_ ≈ 15.3). Throughout this article, results are presented in dimensionless units defined in the “Units” subsection of “Model and Methods.”

We model the condensate as a spherical region of radius *R*_c_ centered at the origin. The condensate acts as a potential well with depth ϵ_c_. This parameter controls the strength of subunit-condensate interactions. We show a plot of the condensate potential for several values of ϵ_c_ in Figure 2H. See Condensate model for additional details. For ϵ_c_ > 0, subunits preferentially partition into the condensate, as characterized by the partition coefficient:

$$K_{c}=\frac{\rho_{1}^{c}}{\rho_{1}^{\mathrm{bg}}}$$ 1

where ρ_1_^c^, ρ_1_^bg^ are the equilibrium concentrations of unassembled subunits in the condensate and background (volume outside the condensate). The condensate occupies a volume *V*_c_, and the background has volume *V*_bg_ = *V* – *V*_c_. We define the condensate volume ratio as *V*_r_ = *V*_c_/*V*_bg_ to characterize the relative sizes of the condensate and background independently of the total volume. Except where otherwise noted, we use *V*_r_ = 5.03 × 10^–3^ for the dodecahedron model and *V*_r_ = 4.20 × 10^–3^ for the icosahedron model. These values are in the typical range for condensates within eukaryotic cells.^92^

### LLPS Promotes Robust Assembly

In a uniform bulk solution, subunit concentration and binding affinity must be carefully tuned to achieve high yields (i.e., the fraction of subunits in complete capsids, *f*_c_, see the “Yields” subsection of “Methods”).^77,84,189,190^ Concentrations and binding affinities larger than their optimal values lead to kinetic traps, such as malformed assemblies or “monomer starvation” (in which many partial assemblies nucleate but do not form complete capsids due to rapid depletion of free monomers^69,76,77,82^ ). On the other hand, concentrations and binding affinities that are too low result in nucleation times that exceed typical time scales of experiments or simulations. Our computational results illustrate these behaviors; in the absence of phase separation (ϵ_c_ = 0) we observe significant yields only in a narrow range of subunit–subunit interaction energies ϵ_ss_ (Figures 3, 4, 5, 6, and Supplementary Movies S1 and S2). We define the yield *f*_c_ = *N*_cap_ρ_cap_/ρ_T_ as the fraction of subunits in complete capsids (including both the condensate and background), with analogous definitions for the condensate *f*_c_^c^ and background *f*_c_^bg^ yields (see eqs 9 and 10 in the “Yields” subsection of “Methods”). Throughout this article we use *yield* to mean *f*_c_, unless otherwise noted.

**Figure 3:**
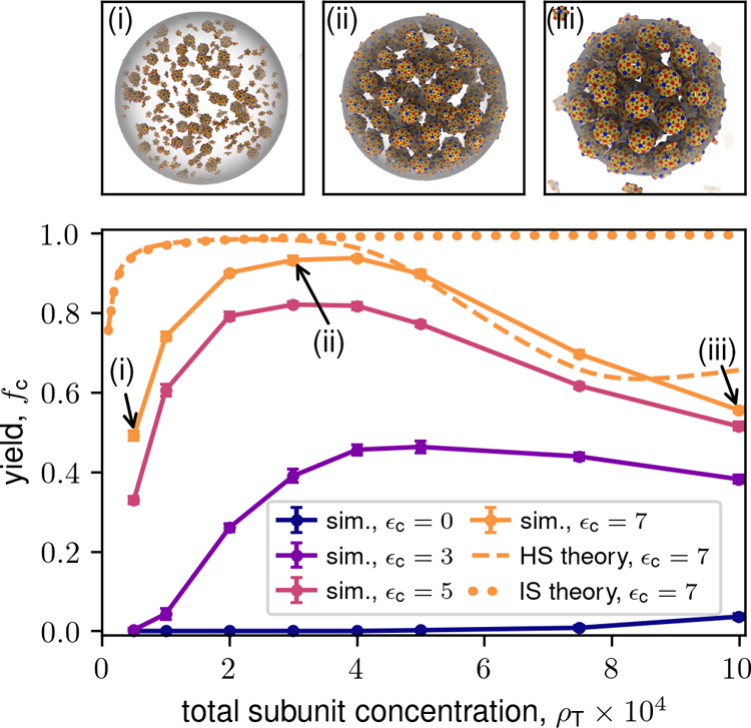
Final capsid yield versus total subunit concentration ρ_T_ for the dodecahedron model for indicated values of ϵ_c_, which controls the partition coefficient *K*_c_. Finite-time simulation yields are shown as symbols with lines. The hard sphere (HS) theory (dashed line) and ideal solution (IS) theory (dotted line) are shown for ϵ_c_ = 7. Markers labeled (i–iii) correspond to snapshots above the plot. Parameters are the subunit–subunit binding energy ϵ_ss_ = 6, condensate volume fraction *V*_r_ = 5.0 × 10^–3^, and final simulation time *t*_F_ = 6 × 10^5^. Except for Figure 4, results are shown for the dodecahedron model.

**Figure 4:**
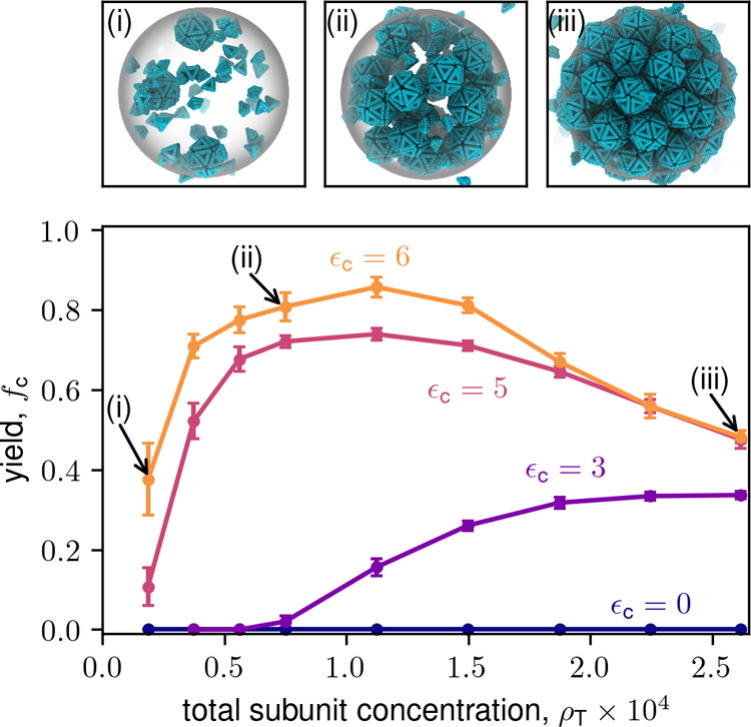
Computational results for final capsid yield versus total subunit concentration for the icosahedron model for indicated values of ϵ_c_. Markers labeled (i–iii) correspond to snapshots above the plot. Parameters are ϵ_ss_ = 6, *V*_r_ = 4.20 × 10^–3^, and *t*_F_ = 10^6^.

**Figure 5:**
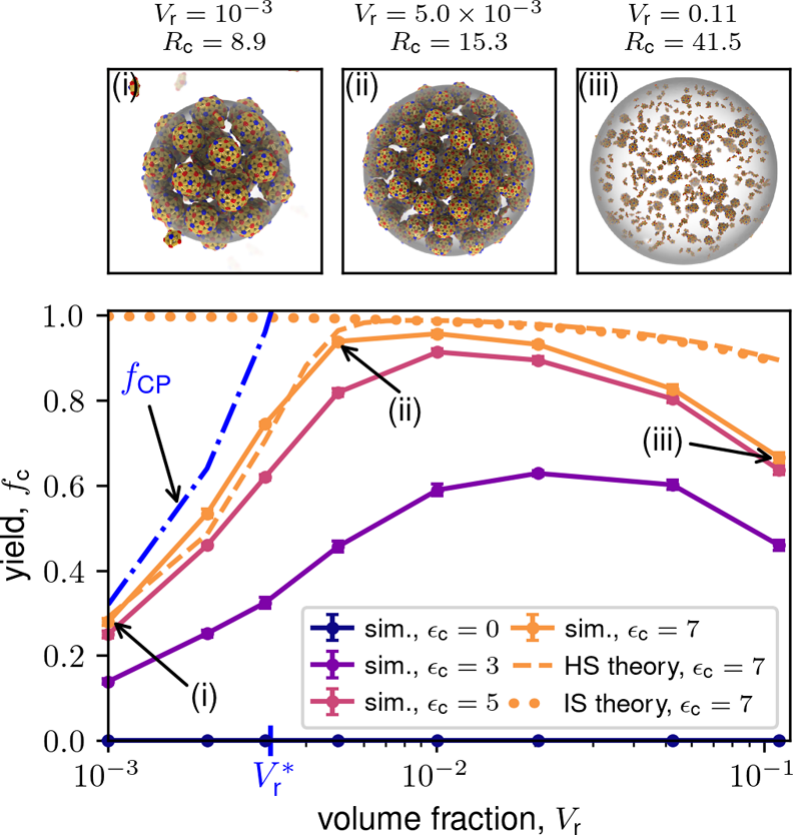
Capsid yield versus condensate volume fraction for indicated values of ϵ_c_ for the dodecahedron model. Finite-time simulation yields are shown as symbols with lines; the hard sphere theory (HS, dashed line) and ideal solution theory (IS, dotted line) predictions are shown for ϵ_c_ = 7. Points labeled with Roman numerals correspond to snapshots above. Supplementary Movie S5 shows the trajectory from which snapshot (iii) was taken. The value *V*_r_^*^, marked in blue on the *x* axis, is the threshold volume fraction below which the close-packed yield theory (see eq 3 and associated discussion in the “LLPS Promotes Robust Assembly” subsection of “Results”) predicts a decline in equilibrium yield. The estimated highest possible yield *f*_CP_ (eq 3, corresponding to spheres with approximately the size of capsids at close-packing density) is shown by the blue dash-dot line. Parameters are ϵ_ss_ = 6, ρ_T_ = 4.00 × 10^–4^, and *t*_F_ = 6 × 10^5^.

**Figure 6:**
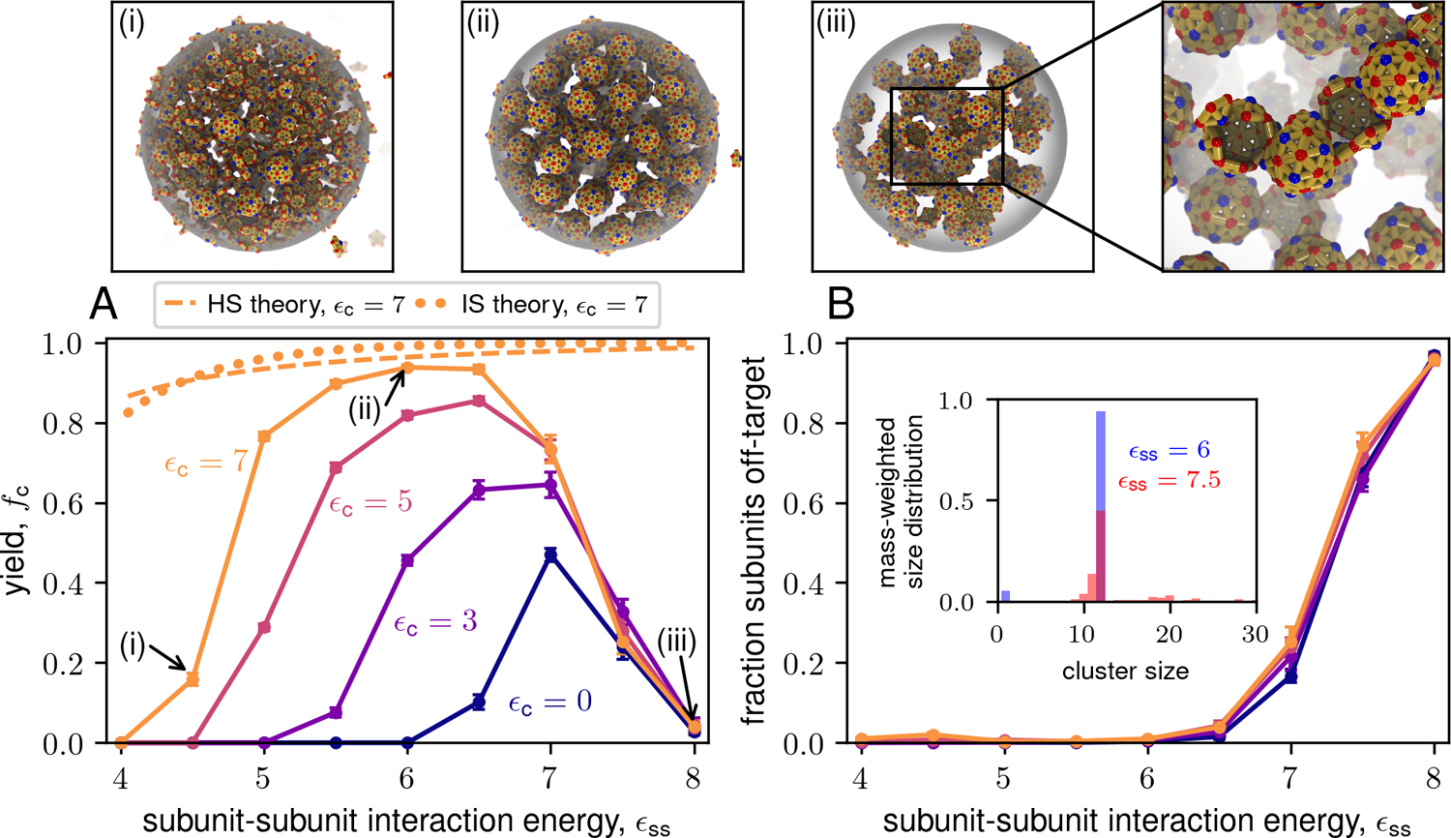
(A) Yield as a function of the subunit binding affinity parameter (ϵ_ss_) at indicated values of ϵ_c_ for the dodecahedron model. Snapshots (i–iii) correspond to the labeled points at three different values of ϵ_ss_ with ϵ_c_ = 7. The inset in (iii) shows an example of a large, malformed assembly consisting of several half-shells bound together. See Supplementary Movie S7 for the trajectory corresponding to snapshot (iii). (B) Fraction of subunits in off-target structures as a function of ϵ_ss_. The inset shows the size distributions at ϵ_ss_ = 6 and ϵ_ss_ = 7.5. Parameters for (A) and (B) are ρ_T_ = 4 × 10^–4^ and *V*_r_ = 5.0 × 10^–3^.

The computational results show that LLPS dramatically improves capsid assembly rates and yields in comparison to bulk assembly (Figures 3, 4, 5, 6 and Supplementary Movies S3, S4, S5). While these trends are qualitatively consistent with rate equation models for assembly coupled to LLPS developed in ref 170, the computational model also exhibits important differences resulting from capsid excluded volume and off-pathway intermediates, which are not accounted for in the previous theory.

#### There Is an Optimal Subunit Concentration due to Capsid Excluded Volume

Figures 3 and 4 show the long (but finite) time yields as a function of total subunit concentration (ρ_T_) for the dodecahedron and icosahedron models, along with representative simulation snapshots. Results are shown for several values of the partition coefficient for a relatively low binding affinity, ϵ_ss_ = 6. While we observe almost no assembly in the absence of LLPS (see also Supplementary Movie S1), except for low yields at high ρ_T_, LLPS leads to high yields over a wide range of concentrations. Essentially all assembly occurs in the condensate, *f*_c_ ≈ *f*_c_^c^ (see Figure S13); our theory (discussed next) indicates that the transition to condensate-dominated assembly occurs for *K*_c_ ≈ 1. However, there is an optimal value of ρ_T_ beyond which yields decrease. For small partition coefficients, the optimal ρ_T_ decreases with increasing partition coefficient, but then seems to saturate; e.g., at ρ_T_ ≈ 3 × 10^–4^ for the dodecahedron model. Note that the saturation value depends on ϵ_ss_ and *V*_r_. The following analysis shows that these trends result from a combination of thermodynamic and kinetic effects.

First, we compare the simulation results against the equilibrium ideal solution theory from ref 170 (see SI Section S3 of this article). The dotted line in Figure 3 shows the approximate equilibrium ideal solution result for the highest partition coefficient (ϵ_c_ = 7), and Figure S1 shows results for different values of ϵ_c_. While qualitatively correct for low ρ_T_, the ideal solution theory fails to capture the decrease in yields for large ρ_T_. The ideal solution rate equation model also predicts high yields in this regime.^170^

While previous simulation results in bulk solution exhibit such decreases in yield due to monomer starvation or malformed structures, we observe that almost all subunits are either monomers or part of assembled capsids for the entire range of ρ_T_ and *K*_c_, with vanishingly few intermediates or malformed structures (see Figure S7). Instead, we can explain the decrease in yield as a *thermodynamic* consequence of subunit excluded volume. For ρ_T_ ≳ 5 × 10^–4^, the concentration of assembled capsids within the condensate is roughly constant (Figure S8), consistent with snapshots which show capsids densely packed within condensates and no capsids in the background (see Figures 3 and 4). Capsids are near the close-packing limit, and hence the condensate cannot accommodate higher capsid concentrations as the total subunit concentration increases. These images are strikingly reminiscent of recent observations of rotavirus capsids closely packed in viroplasms (see Figure 1).

Based on this observation, we developed a theory to predict the equilibrium capsid concentration, which accounts for subunits’ excluded volume by approximating them as hard spheres (see SI Section S3). To briefly summarize the theory, we make the two-state approximation that subunits exist either as monomers or as part of assembled capsids. Equilibrium implies that the chemical potentials of monomers and capsids within each phase are equal, and that the chemical potentials of each species in the condensate and background phases are equal. Assuming that we can model monomer and capsid excluded volume by treating each of these species as effective hard spheres (whose diameters we estimate in SI Section S4), we estimate the chemical potentials using a standard hard sphere equation of state,^191^ and solve the resulting equations self-consistently to obtain the equilibrium concentrations of monomers and capsids in the condensate and background. The hard sphere theory results are shown (dashed line) for ϵ_c_ = 7 in Figure 3 and several values of ϵ_c_ in Figure S1. The theory correctly captures the nonmonotonic dependence of yield on ρ_T_, anticipates that the capsid concentration within the condensate saturates as ρ_T_ increases (Figure S8), and predicts the optimal value of ρ_T_ to within a factor of 2 (≈2 × 10^–4^ in theory vs ≈ 4 × 10^–4^ in simulations). For low ρ_T_ the theoretical yields are above the simulation results, which can be attributed to the fact that the simulation results are finite-time and thus smaller than equilibrium (assembly reactions approach equilibrium asymptotically slowly due to increasing nucleation barriers as subunits are depleted, see Figure S4 and SI Section S5)^84,192^ and that we only roughly estimate the subunit interaction free energy within capsids (see SI Section S4). The theory is less accurate, though still qualitatively good, at high ρ_T_ where capsid concentrations approach the close-packing limit (Figure S8). We do not expect quantitative agreement in this regime because the Carnahan–Starling approximation significantly deviates from real hard-sphere behavior for η ≳ 0.5.^191,193^ Note that the slight increase in the theoretical yield at ρ_T_ ≳ 8 × 10^–4^ arises due to assembly in the background (see Figure S13), which we do not observe in simulations due to nucleation barriers.

Notably, there is a broad range of ρ_T_ over which the total concentration of assembled capsids ρ_cap_ ≈ ρ_cap_^c^ *V*_r_ is roughly constant, although at high enough concentrations (above the bulk critical subunit concentration (ρ_CSC_) capsids will also assemble in the background. Thus, condensates may provide a mechanism to control the total number of assembled capsids, since the number of capsids is bounded by the total condensate volume within this regime. Interestingly, optimal yields occur for capsid concentrations slightly below the close-packing limit, ρ_cap_^c^/ρ_CP_ ≈ 0.6. Assembly is disfavored from reaching the close-packing limit by thermodynamic and kinetic factors: at such high concentrations the excluded volume results in low equilibrium translational entropy for capsids and slow subunit diffusion, which reduces assembly kinetics.

To further assess the effect of capsid excluded volume on yield, Figure S14 shows the equilibrium hard sphere yield (as well as the hard sphere *f*_c_^c^ and *f*_c_^bg^) as a function of ρ_T_ for several values of the capsid effective hard sphere diameter, σ_cap_ (see SI Section S4A for details of how σ_cap_ was estimated). At high concentrations (ρ_T_ ≳ 4 × 10^–4^), which lead to high packing fractions, reducing σ_cap_ increases the yield (though at the highest concentrations, ρ_T_ ≳ 8 × 10^–4^, this effect is compensated by a decrease in the background yield with decreasing σ_cap_). This underscores the potential for condensates to control the number of assembled capsids via excluded volume.

#### There Is an Optimal Condensate Volume Fraction due to Capsid Excluded Volume

The simulation yields also depend nonmonotonically on the condensate volume fraction *V*_r_ (Figure 5), with an optimal value of *V*_r_^*^ that decreases with ϵ_c_, and a sharp decrease in yields and finite-time yield for *V*_r_ ≲ 5 × 10^–3^. This behavior is nearly quantitatively captured by the hard sphere theory for high ϵ_c_, but not by ideal solution theory, which predicts that yields increase monotonically with decreasing *V*_r_ (see Figure S9 for theoretical predictions at different ϵ_c_). The origin of this behavior is evident from simulation snapshots, which show that the condensate becomes highly packed with assembled capsids for high ϵ_c_ and low *V*_r_.

In fact, the yields in this regime and the point corresponding to the steep decline can be qualitatively estimated from a simple sphere packing argument. Although the capsids have dodecahedral symmetry, they have a rounded shape and thus we treat them as effective spheres rather than faceted polyhedra in the following argument. Neglecting free monomers under the assumption of high yield, the maximum number of subunits that can form capsids within a condensate is *N*_max_ ≈ *N*_cap_ *V*_c_ρ_CP_, where ρ_CP_ is the capsid close-packing concentration:

$$\rho_{\mathrm{CP}}=\pi /\left(3\sqrt{2}\right)/\left(4\pi {\left(\sigma_{\mathrm{cap}}/2\right)}^{3}/3\right)\approx 1.28\times 10^{-2}$$ 2

where the capsid excluded volume size σ_cap_ = 5.1 is estimated in SI Section S4A. Thus, below a threshold volume fraction *V*_r_^*^ the total number of subunits exceeds the maximum number in the condensate *N* = *V* ρ_T_ > *N*_max_ and the yield declines (assuming assembly only occurs in the condensate). The threshold is $V_{r}^{*}\approx 1/\left(\frac{N_{\mathrm{cap}}\rho_{\mathrm{CP}}}{\rho_{T}}-1\right)$ , and the corresponding yield for *V*_r_ ≤ *V*_r_^*^ is

$$f_{\mathrm{CP}}=\frac{(V_{r}^{*}{)}^{-1}+1}{V_{r}^{-1}+1}$$ 3

We plot *f*_CP_ in Figure 5 and indicate the value of *V*_r_^*^ (≈2.61 × 10^–3^). The theory qualitatively matches the simulation results, although the decline occurs at slightly larger *V*_r_ in both finite-time simulation results and the equilibrium hard sphere theory. This agreement suggests that the simple packing argument explains the decline in yield at low *V*_r_, but that assembly driving forces are not strong enough to reach complete close-packing. Indeed, Figure S8 shows that capsid concentrations appear to saturate below the close-packing density, reaching ρ_cap_^c^/ρ_CP_ ≈ 0.8 for ϵ_c_ = 7, ρ_T_ = 10^–3^.

#### There Is an Optimal Binding Affinity due to Nucleation Barriers and Malformed Structures

Figure 6A shows the long-time yields as a function of the subunit binding affinity parameter ϵ_ss_ for the dodecahedron model, along with representative simulation snapshots. We observe that LLPS significantly enhances yields, particularly at low ϵ_ss_ for which we observe no assembly in the absence of phase separation. Notably, we even observe higher yields for binding affinities that lead to strong assembly without phase separation (ϵ_ss_ = 7; see Supplementary Movie S2). However, for ϵ_ss_ > 7 there is a sharp decrease in yields for all partition coefficients. In contrast to the case of high subunit concentration, this decline is not captured by the hard sphere theory (shown for ϵ_c_ = 7), indicating that it does not arise due to excluded volume. Instead, the discrepancies arise due to kinetic effects. For low binding affinities, nucleation barriers prevent reaching equilibrium within achievable simulation time scales. Since the nucleation barrier increases with decreasing binding affinity, the simulation results deviate further from equilibrium as ϵ_ss_ decreases. However, as we discuss in the following subsection, “LLPS Enhances Assembly Rates”, LLPS has a significant effect on nucleation time scales.

In contrast, as the binding affinity increases beyond ϵ_ss_ = 7, nucleation barriers are relatively small, but the prevalence of malformed off-pathway structures steadily increases (Figure 6B). The appearance of malformed structures at strong binding affinity values is consistent with previous computational and experimental results from assembly in bulk solution.^69,74,82,84,194–198^ Indeed, for ϵ_ss_ ≥ 7, we observe malformed structures (as well as assembled capsids) in both the background and the condensate (see Supplementary Movies S6 and S7). However, Figure 6 exhibits several notable features that highlight the interplay between malformed structures and LLPS-coupled assembly. First, the fraction of subunits in off-target structures is insensitive to ϵ_c_. This result is unexpected given the high local concentrations of subunits in the condensate, since high concentrations also favor the formation of malformed structures in bulk solution. Second, although the enhancement of yields is much greater for smaller binding affinities, LLPS enhances yields even within the malformed assembly regime. We attribute this enhancement to the ability of LLPS to prevent the monomer starvation trap, as discussed in the following subsection, “LLPS Enhances Assembly Rates”. Third, the structures of malformed assemblies in the condensate differ from those in bulk solution. In bulk, malformed structures usually arise because subunits bind with incorrect geometries and are not significantly larger than well-formed capsids. For example, we commonly observe assemblies with 12 subunits but only 26 bonds. These “danglers”^199^ (Figure S10) form when the twelfth subunit binds in the wrong orientation; the unbinding is slow at high ϵ_ss_. In the condensate we observe fewer danglers (Figure S10), but frequently see partially assembled capsids bound together to form large aggregates (snapshot (iii) in Figure 6 and Supplementary Movie S7). This difference is reflected in a much larger average cluster size for ϵ_ss_ ≥ 7.5 for LLPS-coupled assembly compared to ϵ_c_ = 0 (Figure S11). We attribute these aggregates to the high local concentration of intermediates within the condensate under these conditions; binding of large intermediates to each other frequently leads to malformed structures.^80^

Although previous simulations and experiments show that incorrectly bound subunits and off-pathway intermediates can dissociate and reassemble to form capsids,^69,79,82,172,178,200,201^ the time scales associated with these processes become significantly longer as binding affinities increase. Allowing for intrasubunit flexibility^73,172,201–203^ might facilitate intermediate rearrangement; we will investigate this possibility in future simulations.

### LLPS Enhances Assembly Rates

In addition to enhancing long-time yields, LLPS also significantly increases assembly rates and thus the range of parameters leading to productive assembly at relevant time scales. Figure 7B,E show the yield as a function of time for different values of ϵ_c_ for moderate and large condensate volume fractions respectively (*V*_r_ = 5.0 × 10^–3^ and 0.11), for fixed ϵ_ss_ = 6 and ρ_T_ = 4 × 10^–4^. Figure 8A shows representative simulation snapshots at various times for ϵ_c_ = 7, 3. While there is no assembly without LLPS (ϵ_c_ = 0) at these conditions, we observe rapid and productive assembly with LLPS. Rates increase dramatically with ϵ_c_ for weak partitioning, but begin to level off for ϵ_c_ > 3. More notably, the median assembly time scales τ_1/2_ saturate at a minimum value of τ_1/2_^min^ ≈ 10^5^.

**Figure 7:**
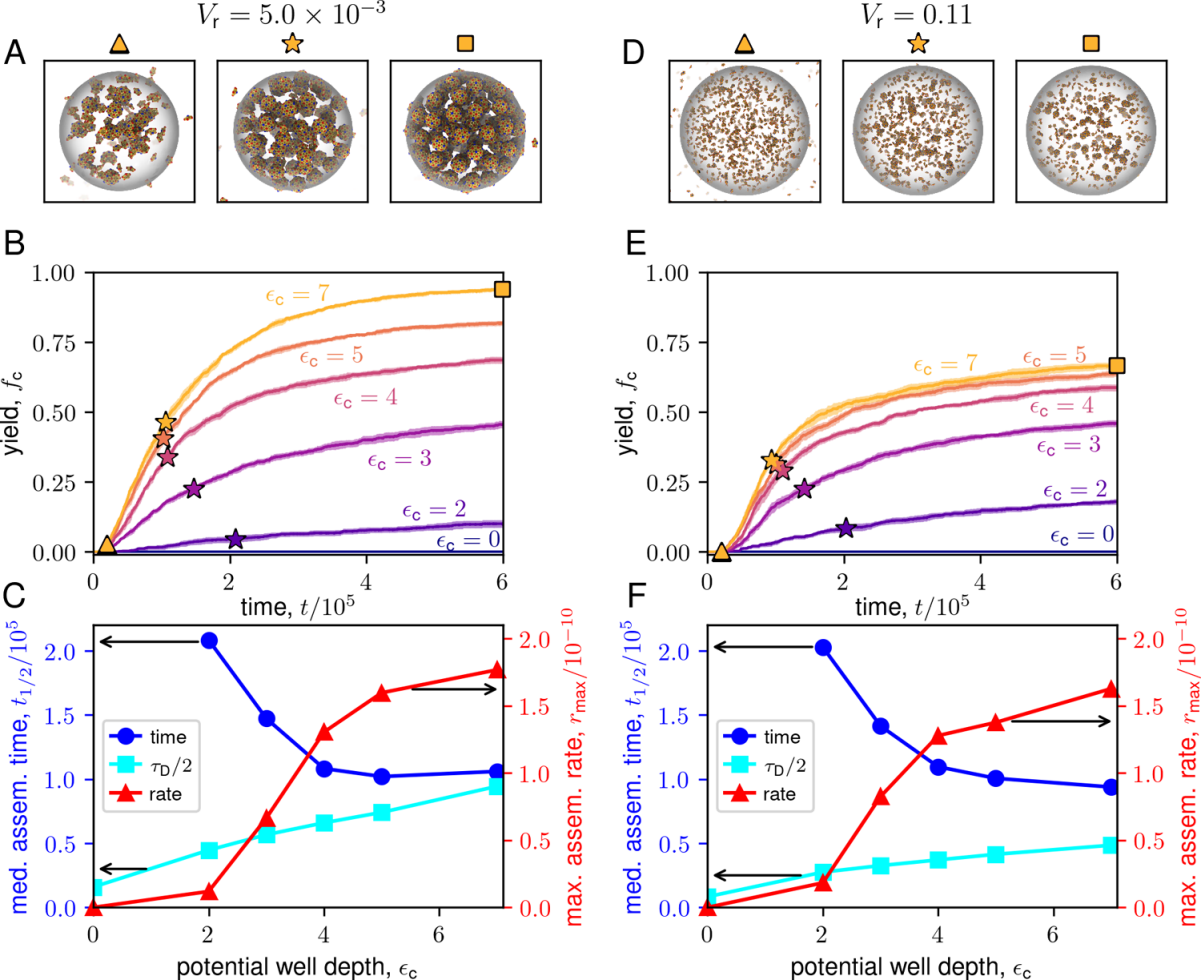
LLPS-coupled assembly kinetics. The left column (panels A–C) shows results for *V*_r_ = 5.0 × 10^–3^, and the right column (panels D–F) shows results for *V*_r_ = 0.11. (A,D) Snapshots from assembly trajectories with ϵ_c_ = 7, corresponding to times *t*/10^5^ = 0.2, 1, and 6, indicated by triangle, star, and square symbols on the plots in (B,E). See Supplementary Movies S4 and S5 for the corresponding trajectories. (B,E) Yield versus time for different values of ϵ_c_. Stars indicate the median assembly times at all ϵ_c_. Parameters for both values of *V*_r_ are ϵ_ss_ = 6, ρ_T_ = 4 × 10^–4^. (C,F) Median assembly time, τ_1/2_ (blue, circles), and maximum assembly rate, *r*_max_ (red, triangles), versus ϵ_c_. The solid cyan line with squares indicates half the diffusion time, τ_D_/2 (eq 4). Arrows indicate the *y* axis to which the lines correspond.

**Figure 8:**
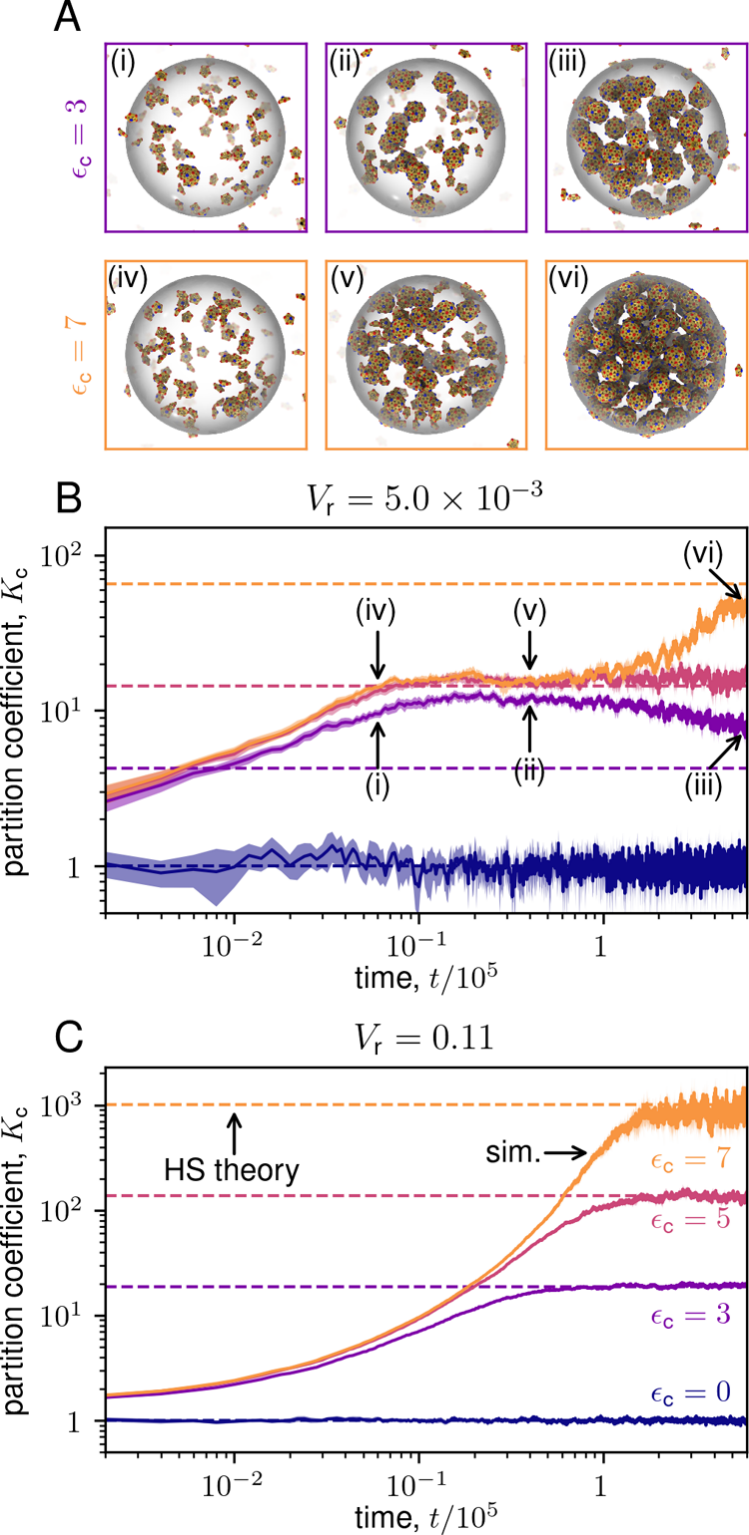
Partitioning of subunits into condensate during assembly. (A) Snapshots from assembly trajectories for *V*_r_ = 5.0 × 10^–3^ and ϵ_c_ = 3 (top) and 7 (bottom). See Supplementary Movies S3 and S4 for segments of the corresponding trajectories. (B) Partition coefficient *K*_c_^meas^(*t*) = ρ_1_^c^(*t*)/ρ_1_^bg^(*t*) measured in simulations as a function of time for indicated values of ϵ_c_ at *V*_r_ = 5.0 × 10^–3^. Solid lines are *K*_c_^meas^(*t*), and dashed lines are the predictions of HS theory. For ϵ_c_ = 3, *K*_c_^meas^ initially rises well above the value of *K*_c_ appropriate for equilibrium between subunits and capsids, instead approaching the equilibrium value of *K*_c_ for no assembly (see Figure 10). (C) *K*_c_^meas^(*t*) for indicated values of ϵ_c_ at *V*_r_ = 0.11.

To further quantify these trends, we plot the median assembly time scales and maximum assembly rates *r*_max_ as a function of ϵ_c_ for moderate and large condensate volume in Figure 7C,F. We estimate the maximum assembly rate by convolving the assembly kinetics with the first derivative of a Gaussian (see Figure S12). We consider the maximum assembly rate as an estimate of the initial nucleation rate because it eliminates the lag time required for a nucleus to grow into a complete capsid.^192^

The observed acceleration of assembly rates with increasing ϵ_c_ and decreasing *V*_r_ is consistent with the prediction from the rate equation theory.^170^ However, the theory fails to capture the saturation of rates at high partitioning strength (see SI Section S6 and Figure S5).

The discrepancy between the ideal solution rate equation and the computational results arises because the interplay between assembly, capsid excluded volume, and subunit diffusion rates leads to a complex dynamics of subunit concentrations within the condensate for high ϵ_c_ and low *V*_r_. Figure 8 shows the partition coefficient *K*_c_^meas^(*t*) = ρ_1_^c^(*t*)/ρ_1_^bg^(*t*) measured in simulations as a function of time for some of the parameter sets in Figure 7. For large condensates (*V*_r_ = 0.11, Figure 8C) subunits steadily partition into the condensate, reaching the equilibrium value of the partition coefficient by *t* = 2 × 10^5^. In contrast, for *V*_r_ = 0.005 (Figure 8B) and high ϵ_c_ ≳ 4, subunit concentrations stall at *K*_c_^meas^ ≈ 10; and then only gradually increase at later times. This is consistent with the observation that median assembly time scales saturate for ϵ_c_ ≳ 4. Intriguingly though, *K*_c_^meas^ overshoots the equilibrium value at early times for ϵ_c_ = 3.

We can understand the results at low *V*_r_ as follows. Simulation trajectories show that assembly occurs extremely rapidly for low *V*_r_ and high partitioning ϵ_c_ ≳ 7 (see snapshot (v) in Figure 8A), depleting subunits fast enough that the subunit flux from diffusion into the condensate (≈4π*RD*ρ_1_^bg^ with *D* = *k*_B_ *T*/γ the subunit diffusion constant) cannot maintain the equilibrium partition coefficient (see Figure 8B). Thus, the condensate reaches a quasi-steady-state where assembly rates are balanced by the subunit flux into the condensate, resembling previous models of assembly in the presence of monomer influx^82,204^ . Then, as the condensate fills with capsids, the high excluded volume further slows subunit entry, leading to an extremely long time scale for subunit densities to reach equilibrium. In contrast, for weaker partitioning ϵ_c_ ≲ 3, nucleation in the condensate is sufficiently slow that subunit partitioning is unimpeded by capsid excluded volume at early times. Thus, subunit concentrations approach the equilibrium partition coefficient in the *absence* of capsid excluded volume (see Figure 10), leading to the apparent overshoot. Then, as assembly proceeds, capsid excluded volume increases, and ρ_1_^c^ decreases toward the equilibrium value.

These considerations show that for sufficiently strong partitioning, reaction rates will eventually be limited by the diffusive flux of subunits into the condensate. We can estimate the characteristic time scale for subunit diffusion into the condensate as (see SI Section S6)­

$$\tau_{D}\approx \frac{V_{c}/(V_{r}+1/K_{c})+f_{c}V_{\mathrm{tot}}}{4\pi R_{c}D}$$ 4

where the first and second terms in the numerator respectively account for diffusion of free subunits and those that eventually form capsids. As shown in Figure 7, τ_D_/2 (the cyan curve, with the factor of $\frac{1}{2}$ because it is the median time) closely matches the minimum median assembly time scale for strong partitioning. Thus, subunit diffusion creates a speed limit on LLPS-facilitated assembly and thus a lower bound on assembly time scales.

#### Implications for Rate Equation Theory

The above considerations show that the ideal solution rate equation theory needs to be extended to account for excluded volume effects as capsids accumulate in the condensate, which lead to reduced partition coefficients (as described in the “Condensate Model” subsection of “Methods”) and slower subunit diffusion with correspondingly slower reaction rates.

In addition, we previously proposed a simple scaling estimate that LLPS accelerates nucleation rates and decreases assembly time scales by a factor (see ref 170 and SI Section S6):

$$\begin{array}{rl} s_{\mathrm{nuc}}\approx V_{r}/(V_{r}+1/K_{c}{)}^{n_{\mathrm{nuc}}} & \mathrm{for}\;V_{r}\ll 1. \end{array}$$ 5

This estimate assumed an ideal solution, a critical nucleus size *n*_nuc_ that is independent of conditions, and that subunit diffusion is fast in comparison to assembly time scales so that the partition coefficient *K*_c_ maintains its equilibrium value. The simulations show that all these assumptions need to be relaxed at strong partitioning. We show in SI Section S6 and Figure S5 that this scaling estimate can be made to qualitatively match simulation results by using the measured values of the partition coefficient *K*_c_^meas^ in *s*_nuc_, and extending the expression for the median assembly time scale to include the diffusion time scale:

$$\tau_{1/2}\approx \tau_{1/2}^{0}/s_{\mathrm{nuc}}+\tau_{D}/2$$ 6

with τ_1/2_^0^ the time scale in the absence of LLPS. The first term in eq 6 gives the median assembly time scale including acceleration by the condensate (*s*_nuc_), while the second term accounts for the diffusion time scale. Further effects that need to be accounted for include the fact that the critical nucleus size can decrease as local subunit concentrations increase and the above-mentioned decrease in reaction rates due to excluded volume.

#### LLPS Enables High Rates by Creating a Buffer of Free Subunits

By spatially localizing assembly within the condensate, LLPS enables extremely high assembly rates while limiting the overall rate of consuming subunits for *V*_r_ ≲ 0.1. Under these conditions, the subunit concentration in the background remains nearly constant for long times, allowing the background solution to act as a “buffer” that steadily supplies free subunits to the condensate. This is a kinetic effect that can avoid the monomer starvation kinetic trap, thus greatly increasing the possible rates and yields of assembly.

We quantify this mechanism by plotting the concentration ρ_1_^bg^ of free subunits in the background as a function of the maximum assembly rate, *r*_max_, for different values of ϵ_c_ over a range of ϵ_ss_ (Figure 9). Each data point in the plot corresponds to a different value of ϵ_ss_ for the indicated ϵ_c_ (shown in different colors). We see that for no LLPS (ϵ_c_ = 0), ρ_1_^bg^ decreases rapidly with increasing *r*_max_ (which is achieved by increasing ϵ_ss_) because rapid nucleation throughout the entire simulation box depletes free subunits. In contrast, with LLPS (*K*_c_ > 1), ρ_1_^bg^ remains fairly close to ρ_T_ for assembly rates that are an order of magnitude higher, thus acting as a buffer. However, even with LLPS there is eventually a binding affinity above which the maximum rate decreases, due to assembly in the background solution, formation of malformed structures, or overly rapid assembly within the condensate.

**Figure 9:**
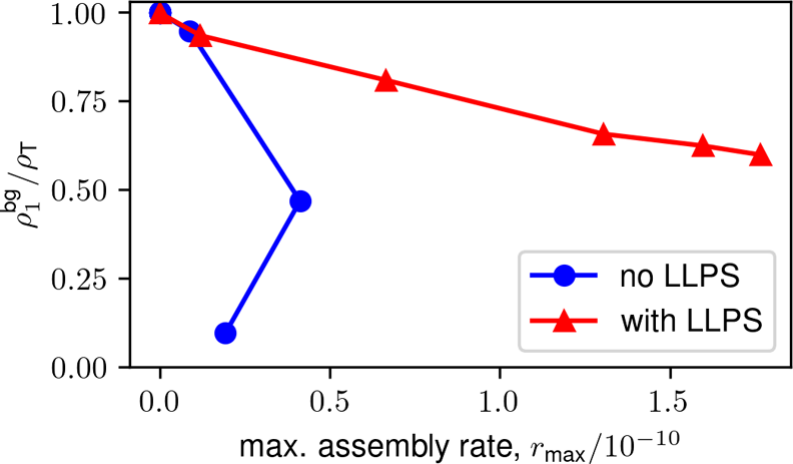
Bulk solution acts as a buffer of free subunits with LLPS. The plot shows the background concentration ρ_1_^bg^ (normalized by the total concentration ρ_T_) as a function of the maximum assembly rate both with (red, triangles) and without (blue, circles) LLPS. Each data point for “no LLPS” corresponds to a different value of ϵ_ss_, which ranges from 4 ≤ ϵ_ss_ ≤ 8, and ϵ_c_ = 0. Each data point for “with LLPS” corresponds to a different ϵ_c_, with ϵ_ss_ = 6.

## Conclusions

While these results are qualitatively consistent with previous rate equation models,^169,170^ the particle-based simulations exhibit important differences due to excluded volume effects and aberrant off-pathway structures that are not accounted for in ideal solution theory and simplified rate equations. In particular, there is an optimal total subunit concentration ρ_T_ and condensate volume fraction *V*_r_. For higher total subunit concentrations or lower condensate volumes, yields are limited by capsid excluded volume in the condensate. We developed an equilibrium theory for capsid assembly that includes these excluded volume effects, which agrees well with the simulation results. For higher-than-optimal binding affinity values, yields are suppressed by malformed off-pathway structures, independent of the subunit partition coefficient *K*_c_. However, malformed structures form large aggregates within the condensate, while they tend to be on the order of the capsid size in bulk. In addition, we find that assembly rates are limited at high partition coefficients by the rate of subunit diffusion into the condensates. Rates are then further reduced by slow subunit diffusion once capsid excluded volume in the condensate becomes large. The latter effect is consistent with previous theoretical models.^167,168^ We show how the rate equation and associated scaling estimate for LLPS-facilitated assembly rates and time scales^170^ can be extended to include these effects.

### Diffusion Sets a Speed Limit for LLPS-Facilitated Assembly

For sufficiently strong partition coefficients, leading to high local subunit concentrations within the condensate, assembly rates can become limited by the diffusive flux of subunits into the condensate. The associated diffusive time scale sets a lower bound on the time scale for LLPS-facilitated assembly.

### Testing in Experiments

Our predictions on the dependence of yield and assembly rates on subunit concentrations and binding affinity values could also be tested in both biological and synthetic experiments in which self-assembly occurs within phase-separated condensates. In addition to viruses—the system that most closely motivates this work—our predictions could also be tested other cases described in the introduction (e.g., clathrin cages, actin filaments, and assemblies within neuronal synapses^103–108^ ). However, these predictions may be more readily tested in in vitro experiments which allow greater control over parameter values and condensate sizes, such as biomolecular assemblies,^92,102,117,205^ or recently developed DNA origami subunits that form capsids and tubules.^28,29,31,32,206^

The computational prediction that capsids fill phase-separated compartments to near close-packing densities under optimal assembly conditions are evocative of recent observations from cells infected with rotavirus.^116,123^ It would be of interest to investigate whether similar capsid arrays are observed in other viruses, what conditions give rise to such arrays, and more generally how capsid packing densities depend on parameter values.

### Implications for Biology and Synthetic Assembly Systems

The ability of LLPS to increase assembly rates could be crucial for viruses to assemble before detection by host immune systems, and thus may be one reason why many viral systems form phase-separated compartments during their lifecycles. Further, while viruses have limited control over conditions within the host cell, they can control the partition coefficient and condensate volume. Faster assembly rates and enhanced control may also explain why many other biological assembly processes occur within condensates. In addition, the computational results show that there is a broad range of concentrations over which capsid concentrations within the condensate are nearly constant. Thus, by controlling the condensate volume, viruses can control the total number of assembled capsids. This might also provide a mechanism to control the timing of assembly—additional assembly would occur whenever capsids are released from the condensate.

Our results suggest that accounting for effects of condensates may be important to link in vitro results to virus assembly in the biological setting. For example, we observe condensate-coupled assembly at significantly lower subunit–subunit binding affinities and concentrations compared to bulk assembly due to the stabilization and localization of subunits by the condensate (see Figures 3, 4, and 6). Thus, binding affinities for viruses that exploit condensates could be weaker than the ranges estimated from bulk in vitro experiments.

Importantly, these same considerations suggest that LLPS could provide a means to enable rapid, robust assembly in human-engineered systems. In addition, our study identifies a route for efficient bottom-up assembly of highly monodisperse arrays of assemblies, which could have important optoelectronic applications. In contrast to previous approaches that can only assemble macroscopic crystals of capsids,^207–209^ condensate-coupled assembly enables precisely controlling the size of the crystalline arrays.

### Outlook

The excluded volume effects identified in this work could be, at least approximately, accounted for in rate equation models following the approach that we have used for the equilibrium theory. Moreover, the theory can be applied to other assembly reactions occurring at high concentrations, such as the assembly of the mature HIV capsid.^210–222^ Although our computational model accounts for this and other effects that were neglected in previous rate equation models, we have only implicitly modeled the condensate and assumed that the condensate phase coexistence is independent of subunit concentration. A natural next step is to explicitly model the constituents that form the condensate. This will allow studying the effect of subunits on condensate phase separation as well as additional effects that may arise from the excluded volume of condensate components. For example, expansion of the condensate could reduce excluded volume effects, although the extent of condensate expansion would be limited by the phase coexistence conditions. We have also not considered actively driven condensates.^129–136^ While we are not aware of strong effects from activity within viral condensates, active flows^223–227^ could influence assembly behaviors in other types of condensates. Finally, condensate-mediated assembly bears some similarities to enhanced protein crystal nucleation near a fluid–fluid critical point, in which transient density fluctuations locally concentrate proteins and thus lower the crystal nucleation barrier.^228^ It would be interesting to investigate possible connections between protein crystal nucleation and assembly in viral condensates, particularly those (like SARS-CoV-2 ^229^ and Ebola^230^) in which the capsid proteins themselves are the major condensate component (scaffold).

## Methods

### Subunit Models

We use molecular dynamics to simulate *N* subunits in a cubic box with edge-length *L* and volume *V* = *L* ^3^ (subject to periodic boundary conditions) at a temperature *T*. We perform our simulations using the GPU-accelerated HOOMD-blue package^231^ (v2.9.0 for the icosahedron model, v4.6.0 and v4.8.0 for the dodecahedron model). To reduce the computational cost of simulating high-frequency vibrational motions, both the dodecahedron and icosahedron models represent capsid subunits as rigid bodies.

To focus on the interplay between assembly and LLPS, in this study we consider empty capsids (containing no nucleic acid). However, in systems where the nucleic acid plays an active role in capsid assembly there may be additional factors controlling assembly behavior and alternative assembly pathways.^5,8,174,187,188,232–236^

#### Dodecahedron Model

In the dodecahedron model (Figure 2A–C), pentagonal subunits (Figure 2A) with circumradius *l*_0_ assemble into dodecahedral capsids (Figure 2C).The subunits consist of a top (‘T’) pseudoatom that has repulsive interactions with other ‘T’ pseudoatoms, a bottom (‘B’) pseudoatom that has repulsive interactions with ‘T’ pseudoatoms to help prevent subunits from binding with upside-down configurations, and five attractor (‘A’) pseudoatoms located at the vertices of the pentagon that account for subunit–subunit attractions ((Figure 2B and SI Section S1). The ‘T’ and ‘B’ interactions are represented by a WCA-like potential.^237^ The ‘A’ pseudoatoms interact via a Morse potential, for which the well-depth parameter ϵ_ss_ sets the subunit–subunit binding affinity, which we estimate in SI Section S4.

#### Icosahedron Model

In the icosahedron model (Figure 2D–F), triangular subunits (Figure 2D) of diameter ≈ 3σ (where σ is the diameter of the pseudoatoms comprising each subunit) assemble assemble into *T* = 1 icosahedral capsids (Figure 2F).^31^ These subunits consist of 45 excluder pseudoatoms that account for excluded volume via a WCA potential, and six attractor pseudoatoms which bind to complementary attractor atoms via a Lennard-Jones potential with well depth ϵ_ss_ (see Figure 2B and SI Section S1). We describe both the dodecahedron and icosahedron models in detail in the Supporting Information (SI) Section S1.

### Condensate Model

We model the condensate as a spherical region of radius *R*_c_ centered at the origin. The condensate imposes a spherically symmetric potential given by

$$u_{c}(r)=\{\begin{array}{ll} -\epsilon_{c}, & r<R_{c}\\ -\epsilon_{c}(2e^{-\alpha_{C}(r-R_{c})}-e^{-2\alpha_{C}(r-R_{c})}), & r\geq R_{c} \end{array}$$ 7

where *r* is the distance from the origin, ϵ_c_ sets the depth of the potential well, and α_C_ is a parameter that controls how rapidly the potential decays to zero as *r* increases beyond *R*_c_. We set α_C_ = 10 *l*_0_^–1^ (10σ^–1^ for the icosahedron model), which ensures that the potential goes to zero over a length scale α_C_^–1^ ≪ *R*_c_ while suppressing unphysically large forces at the condensate boundary. We show a plot of *u*_c_(*r*) for several values of ϵ_c_ in Figure 2H.

The well-depth ϵ_c_ controls the strength of subunit-condensate interactions. For ϵ_c_ > 0, subunits preferentially partition into the condensate, as characterized by the partition coefficient:

$$K_{c}=\frac{\rho_{1}^{c}}{\rho_{1}^{\mathrm{bg}}}$$ 8

where ρ_1_^c^, ρ_1_^bg^ are the equilibrium concentrations of unassembled subunits in the condensate and background (volume outside the condensate). In the ideal solution (IS) limit (for packing fractions $\eta =\frac{\pi }{6}\rho l_{0}^{3}≲0.1$ ) the partition coefficient is related to the well depth by *K*_c_^IS^ = *e* ^βϵ_c_^, and assembly will significantly increase partitioning of larger structures, since the driving force for a cluster with *n* subunits to partition into the condensate is ∝ *n*ϵ_c_. However, for higher concentrations excluded volume effects will reduce the partition coefficient, particularly once capsids assemble due to their large excluded volume. We account for excluded volume effects of monomers alone (i.e., before assembly occurs) as well as in the presence of assembly by modeling subunits and capsids as effective hard spheres using the Carnahan–Starling equation of state^193,238^ and its extension to binary mixtures^191^ (see Figures 10, S6, and SI Section S2). See the Appendix for additional discussion of how *K*_c_ depends on ϵ_c_. A snapshot of a condensate containing both monomeric subunits and capsids is shown in Figure 2G.

**Figure 10:**
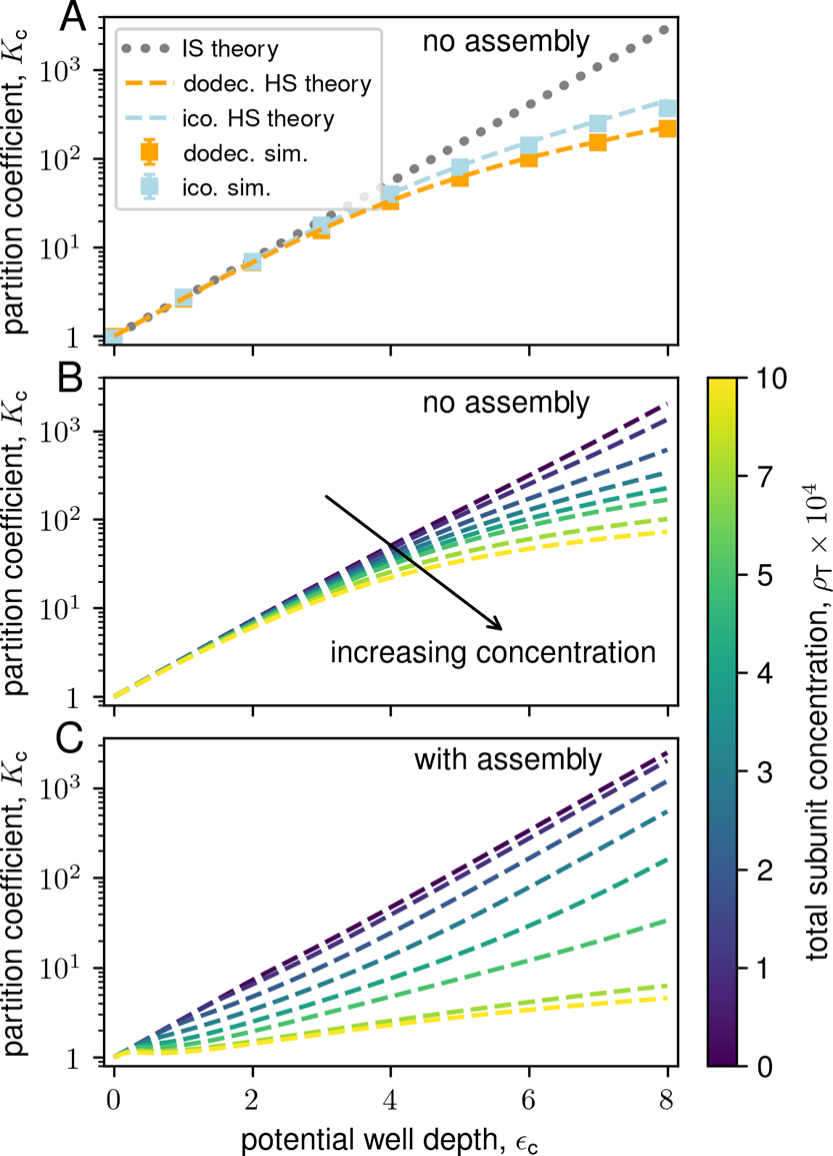
Effects of excluded volume on the partition coefficient, with and without assembly. (A) Partition coefficient *K*_c_ versus condensate potential well depth ϵ_c_ with no assembly (i.e., no subunit–subunit attractions, ϵ_ss_ = 0). The gray dotted line represents the ideal solution (IS) theory. Orange and light blue represent results for the dodecahedron and icosahedron models, respectively, at concentrations ρ_T_ = 4 × 10^–4^ (dodecahedron) and ρ_T_ = 7.5 × 10^–5^ (icosahedron). The dashed lines represent the hard sphere (HS) theory using the Carnahan–Starling equation of state (see Section S2), and the square markers represent results from simulations with ϵ_ss_ = 0 (error bars are smaller than the size of the plotted points). In both cases, ideal solution theory breaks down when packing fractions in the condensate exceed η ≳ 10*%* (Figure S6). (B) Hard sphere theory results for the dodecahedron model without assembly over a range of concentrations. At the highest well depth and subunit concentration, excluded volume causes *K*_c_ to decrease by over an order of magnitude compared to ideal solution theory. (C) Hard sphere theory results for the dodecahedron model with assembly over a range of concentrations. At high concentrations, excluded volume causes *K*_c_ to decrease by orders of magnitude compared to ideal solution theory predictions.

### Units

In both the dodecahedron and icosahedron models, the thermal energy *k*_B_ *T* (where *k*_B_ is Boltzmann’s constant) serves as our unit of energy and the subunit mass *m* is the unit of mass. In the dodecahedron model, *l*_0_ is the unit length, and $t_{0}≡l_{0}\sqrt{m/k_{B}T}$ is the unit time. For the icosahedron model, σ is the unit length and $t_{0}≡\sigma \sqrt{m/k_{B}T}$ is the unit time. We report all quantities in terms of these units (energies in *k*_B_ *T*, lengths in *l*_0_ for the dodecahedron model and σ for the icosahedron model, and times in *t*_0_).

### Yields

To measure the extent of capsid assembly, we define the capsid *yield* as the fraction of subunits in complete capsids, *f*_c_:

$$f_{c}=N_{\mathrm{cap}}\rho_{\mathrm{cap}}/\rho_{T}$$ 9

where *N*_cap_ is the number of subunits comprising a complete capsid (12 for the dodecahedron model, 20 for the icosahedron model) and ρ_cap_ is the total concentration of capsids (in both the background and condensate). We also define the yields of capsids in the condensate (*f*_c_^c^) and background (*f*_c_^bg^) as

$$\begin{array}{c} f_{c}^{c}=\frac{N_{\mathrm{cap}}\rho_{\mathrm{cap}}^{c}}{\rho_{T}}\frac{V_{r}}{1+V_{r}}\\ f_{c}^{\mathrm{bg}}=\frac{N_{\mathrm{cap}}\rho_{\mathrm{cap}}^{\mathrm{bg}}}{\rho_{T}}\frac{1}{1+V_{r}} \end{array}$$ 10

A complete capsid for the dodecahedron or icosahedron model has 12 or 20 subunits respectively, and each subunit forms the maximum number of bonds (as defined in the “Simulation Details” subsection next) with its neighbors, 5 or 3 respectively.

### Simulation Details

For the dodecahedron model, we use *N* = 1200 subunits, and vary the box size from *L* = 288.5 to 106.3 to control the concentration over the range ρ_T_ ∈ [5 × 10^–5^, 10^–3^]. For the icosahedron model, we use *L* = 200 and *N* = 149 to 2092 to achieve ρ_T_ ∈ [1.8 × 10^–5^, 2.6 × 10^–4^].

Trajectories are initialized with subunits placed in the box at random, nonoverlapping positions. We then run Langevin dynamics for *t*_F_ = 10^6^ (1.2 × 10^8^ time steps with time step Δ*t* = 5 × 10^–3^ for the dodecahedron model and 2 × 10^8^ time steps with Δ*t* = 5 × 10^–3^ for the icosahedron model) unless noted otherwise. All reported results were obtained by averaging over 10 (dodecahedron) or 5 (icosahedron) independent trajectories for each parameter set (ϵ_ss_, ϵ_c_, and ρ_T_). Error bars represent twice the standard error of the mean (approximately a 95% confidence interval).

We used our open-source Python library SAASH^239^ to analyze trajectories and calculate the yield of complete capsids. The library provides functions to identify fully assembled capsids as well as arbitrarily sized clusters. For cluster identification, we consider two dodecahedron subunits to be bonded (and hence part of the same cluster) if the centers of the edges of two subunits are within 0.3 of each other; we consider two icosahedron subunits to be bonded if both pairs of attractor pseudoatoms on the two subunits are within 1.3 of each other. Additional Python scripts used in this work have been deposited on Github at the following URL: https://github.com/Layne28/implicit-condensate.

## Supplementary Material





## Data Availability

Our simulation and analysis scripts are available on Github: https://github.com/Layne28/implicit-condensate. Additionally, postprocessed trajectory data, Mathematica notebooks for theoretical calculations, and figure-generating scripts are hosted on the Open Science Framework OSFHome (https://osf.io/hq2y8/).

## Appendix: Partition coefficient versus ϵ_c_

Figure 10 shows the partition coefficient *K*_c_ as a function of potential well depth ϵ_c_ for both capsid models at a single concentration with no assembly, and for the dodecahedron model with and without assembly over a range of concentrations.

## Notes

Detailed descriptions of capsid models and molecular dynamics simulations; detailed calculations of partition coefficients within ideal solution and hard sphere theories; equilibrium assembly theory calculations; estimates for theoretical model parameters; yield versus subunit–subunit interaction energy at different simulation times; scaling theory for assembly kinetics; additional supplementary figures; and descriptions of supplementary movies, including simulation parameters (PDF)
Trajectory showing no assembly without LLPS; capsid assembly trajectory without LLPS; capsid assembly trajectory with LLPS for relatively weak subunit partitioning; capsid assembly trajectory with LLPS for relatively stong subunit partitioning; capsid assembly trajectory with LLPS for a relatively large condensate volume fraction; capsid assembly trajectory without LLPS at high subunit binding affinity, exhibiting malformed structures; and capsid assembly trajectory with LLPS at high subunit binding affinity, exhibiting malformed structures (ZIP)
