# Supplementary material for: Computer Simulations Show That Liquid–Liquid Phase Separation Enhances Self-Assembly
Source: ACS Nano. 2025 Aug 9;19(33):30275–91. doi: 10.1021/acsnano.5c08120 (PMC12392740; doi:10.1021/acsnano.5c08120)
Supplement: Supplementary file 1 [file nn5c08120_si_001.pdf]

Supplementary Information: Computer simulations show that liquid-liquid phase separation enhances self-assembly

Layne B. Frechette,* Naren Sundararajan,* Fernando Caballero, Anthony Trubiano, and Michael F. Hagan†
Martin Fisher School of Physics, Brandeis University, Waltham, Massachusetts 02453, USA
(Dated: August 5, 2025)

CONTENTS

S1. Model description 1
A. Dodecahedral capsid model 1
B. Icosahedral capsid model 2
C. Dynamics 3

S2. Partition coefficient calculation 3
A. Ideal solution theory 3
B. Hard sphere theory 3

S3. Equilibrium assembly theory 4
A. Ideal solution theory 4
B. Hard sphere theory 5

S4. Estimating parameters for the theoretical model 6
A. Estimating σ_{cap} 6
B. Estimating f_{capsid} 6

S5. Yield vs ϵ_{ss} at different times 7

S6. Scaling theory for assembly kinetics 8

S7. Additional supplementary figures 10

S8. Movie descriptions 15

References 15

S1. MODEL DESCRIPTION

A. Dodecahedral capsid model

In the dodecahedron model, each subunit consists of seven pseudoatoms of three different types. Five attractor pseudoatoms ('A'), which facilitate subunit-subunit binding, are located at the vertices of of a regular pentagon with circumradius l_0, which we take as the unit of length for this model. 'A' pseudoatoms on different subunits interact via a Morse potential:

$$u_{\text{AA}}(r) = \begin{cases} \epsilon_{\text{ss}} \left[e^{-2\alpha(r-r_0)} - 2e^{-\alpha(r-r_0)} \right] - \epsilon_0, & r \leq 2l_0 \\ 0, & r > 2l_0 \end{cases} \tag{S1}$$

where r is the distance between 'A' pseudoatoms, $r_0 = l_0/5$ is the distance at which the potential energy is minimized, $\alpha = 2.5/r_0$ sets the width of the potential well, and $\epsilon_0 = \epsilon_{\text{ss}} \left[e^{-2\alpha(2l_0-r_0)} - 2e^{-\alpha(2l_0-r_0)} \right]$ is chosen so that the potential

* These two authors contributed equally.
† hagan@brandeis.edu

goes to zero continuously at $r = 2l_0$. The parameter ϵ_{ss} sets the depth of the potential well and hence controls the subunit-subunit binding affinity. Additionally, each subunit contains one top pseudoatom ('T') and one bottom pseudoatom ('B') located at $z = \pm l_0/2$ relative to the centroid of the pentagon, which lies in the xy plane in the body-fixed frame. 'T' pseudoatoms interact via a purely repulsive potential:

$$u_{\mathrm{TT}}(r) = \mathcal{L}(r; \sigma_{\mathrm{TT}}, \epsilon_{\mathrm{TT}}), \tag{S2}$$

where:

$$\mathcal{L}(r; \sigma, \epsilon) = \begin{cases} 4\epsilon \left[\left(\frac{\sigma}{r}\right)^{12} - \left(\frac{\sigma}{r}\right)^{6} \right], & r \leq \sigma \\ 0, & r > \sigma. \end{cases} \tag{S3}$$

We choose $\epsilon_{\mathrm{TT}} = 1$ and $\sigma_{\mathrm{TT}} = 2.1l_0$, which encourages a subunit-subunit binding angle consistent with a dodecahedron. 'B' pseudoatoms interact with 'T' pseudoatoms via a similarly repulsive potential:

$$u_{\mathrm{TB}}(r) = \mathcal{L}(r; \sigma_{\mathrm{TB}}, \epsilon_{\mathrm{TB}}), \tag{S4}$$

where $\epsilon_{\mathrm{TB}} = \epsilon_{\mathrm{TT}}$ and $\sigma_{\mathrm{TB}} = 1.8l_0$. The 'T'-'B' interactions help prevent "upside down" assembly, by discouraging subunit-subunit binding that results in 'T' and 'B' pseudoatoms on different subunits being adjacent. Subunits and their interactions are depicted in Fig. 2A,B, and a dodecahedral capsid resulting from the assembly of twelve subunits is shown in Fig. 2C. SI Section S4B provides an estimate of the subunit-subunit binding affinity and capsid free energy as a function of ϵ_{ss}.

B. Icosahedral capsid model

The icosahedron model was previously developed to study the assembly of DNA origami capsids [1, 2]. The subunit shape and the interactions between the constituent pseudoatoms are designed such that the minimum energy configuration of a collection of twenty subunits is an icosahedral shell (see Fig. 2F). Subunits consist of 45 purely repulsive excluder pseudoatoms and six attractor pseudoatoms. As in the dodecahedron model, subunit-subunit binding affinities are governed by the parameter ϵ_{ss}. However, unlike the dodecahedron model, not all attractor atoms interact with each other equally. Instead, each attractor pseudoatom within a subunit has a distinct type (represented as different colors in Fig. 2D,E). Pairs of complementary attractor atoms attract each other, while non-complementary attractor atoms interact only via excluded volume. This prevents subunits from binding "upside down" (thus it has a similar role to the repulsive 'T'-'B' interactions in the dodecahedron model). Complementary attractor pairs are listed in Table S1.

TABLE S1. Complementary attractors (1=complementary, 0=non-complementary)

	blue	red	gray	orange	yellow	tan
blue	0	1	0	1	0	1
red	1	0	1	0	1	0
gray	0	1	0	1	0	1
orange	1	0	1	0	1	0
yellow	0	1	0	1	0	1
tan	1	0	1	0	1	0

Excluder pseudoatoms and non-complementary attractor pseudoatoms interact via a Weeks-Chandler-Anderson (WCA) potential [3]:

$$u_{\mathrm{WCA}}(r) = \begin{cases} 4\epsilon_{\mathrm{ss}} \left[\left(\frac{\sigma}{r}\right)^{12} - \left(\frac{\sigma}{r}\right)^{6} + \frac{1}{4} \right], & r < 2^{1/6}\sigma \\ 0, & r \geq 2^{1/6}\sigma \end{cases} \tag{S5}$$

where r is the distance between pseudoatoms and σ is the pseudoatom diameter, which we take as the unit of length for this model. Complementary attractor pseudoatoms interact via a Lennard-Jones potential:

$$u_{\mathrm{ss}}(r) = \begin{cases} u_{\mathrm{LJ}}(r) - u_{\mathrm{LJ}}(r_{\mathrm{cut}}), & r < r_{\mathrm{cut}} \\ 0, & r \geq r_{\mathrm{cut}} \end{cases} \tag{S6}$$

where $u_{\rm LJ}$ is given by

$$u_{\rm LJ}(r) = 4\epsilon_{\rm ss}\left[\left(\frac{\sigma}{r}\right)^{12} - \left(\frac{\sigma}{r}\right)^{6}\right]. \tag{S7}$$

and $r_{\rm cut} = 3\sigma$. Two interacting subunits are depicted in Fig. 2E.

C. Dynamics

Subunits (labeled $i = 1, ..., N$) evolve in time via Langevin dynamics (see the HOOMD-blue documentation, [4], and Eq. 3.37 in Ref. [5]):

$$m\frac{\mathrm{d}\mathbf{v}_i}{\mathrm{d}t} = \mathbf{F}_{\mathrm{C},i} - \gamma\mathbf{v}_i + \mathbf{F}_{\mathrm{R},i}, \tag{S8a}$$

$$\mathbf{v}_i = \frac{\mathrm{d}\mathbf{r}_i}{\mathrm{d}t}, \tag{S8b}$$

$$\mathbf{I}\frac{\mathrm{d}\boldsymbol{\omega}_i}{\mathrm{d}t} = \boldsymbol{\tau}_{\mathrm{C},i} - \gamma_{\mathrm{r}}\boldsymbol{\omega}_i + \boldsymbol{\tau}_{\mathrm{R},i} \tag{S8c}$$

$$\frac{\mathrm{d}}{\mathrm{d}t}\begin{pmatrix} q_{0,i} \\ q_{1,i} \\ q_{2,i} \\ q_{3,i} \end{pmatrix} = \frac{1}{2}\begin{pmatrix} q_{0,i} & -q_{1,i} & -q_{2,i} & -q_{3,i} \\ q_{1,i} & q_{0,i} & -q_{3,i} & q_{2,i} \\ q_{2,i} & q_{3,i} & q_{0,i} & -q_{1,i} \\ q_{3,i} & -q_{2,i} & q_{1,i} & q_{0,i} \end{pmatrix}\begin{pmatrix} 0 \\ \omega_{x,i} \\ \omega_{y,i} \\ \omega_{z,i} \end{pmatrix} \tag{S8d}$$

Here m is the mass and $\mathbf{I}$ is the (body-fixed and hence diagonal) inertia tensor of a subunit; γ and γ_{r} are translational and rotational friction constants; $\mathbf{r}_i$ and $\mathbf{v}_i$ are the center of mass position and velocity; $\mathbf{r}_i$ and $\mathbf{v}_i$ are the center of mass position and velocity; $\mathbf{q}_i$ is the orientation (represented as a quaternion) and $\boldsymbol{\omega}_i$ is the body-fixed angular velocity; $\mathbf{F}_{\mathrm{C},i}$ and $\boldsymbol{\tau}_{\mathrm{C},i}$ are the conservative force and torque that come from the potential energy U, which includes a sum of pairwise interactions between pseudoatoms (Eqs. S1, S2, and S4 for the dodecahedron model; Eqs. S5 and S6 for the icosahedron model), a sum over single-pseudoatom energies due to the condensate potential (Eq. 7)), and constraint forces that ensure rigid body motion. The quantities $\mathbf{F}_{\mathrm{R},i}$ and $\boldsymbol{\tau}_{\mathrm{R},i}$ are the random (thermal) force and torque, which have zero mean and variances given by:

$$\langle \mathbf{F}_{\mathrm{R},i}(t) \cdot \mathbf{F}_{\mathrm{R},j}(t')\rangle = 6k_{\mathrm{B}}T\gamma\delta(t-t')\delta_{ij} \tag{S9a}$$

$$\langle \boldsymbol{\tau}_{\mathrm{R},i}(t) \cdot \boldsymbol{\tau}_{\mathrm{R},j}(t')\rangle = 6k_{\mathrm{B}}T\gamma_{\mathrm{r}}\delta(t-t')\delta_{ij}. \tag{S9b}$$

We set $\gamma = 10$ and $\gamma_{\mathrm{r}} = 4\gamma/3$, which makes the dynamics effectively overdamped (consistent with the viscous interior of a cell). Within HOOMD-blue [4], we use the velocity Verlet algorithm to solve Eqs. S8a, S8b, and the similarly symplectic integrator of Kamberaj et al. [6] to solve Eqs. S8c, S8d. We employ a timestep $\Delta t = 5 \times 10^{-3}$.

S2. PARTITION COEFFICIENT CALCULATION

A. Ideal solution theory

For sufficiently low concentrations of subunits within the condensate, ideal solution theory gives $K_{\mathrm{c}}^{\mathrm{IS}} = \frac{\rho_1^{\mathrm{c}}}{\rho_1^{\mathrm{bg}}} = e^{\beta\epsilon_{\mathrm{c}}}$. For subunit densities and partition coefficients that lead to low packing fractions η within the condensate $\eta \lesssim 10\%$ this theory predicts the measured partition coefficient with reasonable accuracy (Fig. 10 in main text). For larger values, we observe significant deviations between ideal solution theory and simulation results without assembly, in which we set $\epsilon_{\mathrm{ss}} = 0$ for attractor pseudoatoms (but keep $\epsilon_{\mathrm{ss}} = 1$ for icosahedron model excluder pseudoatoms).

B. Hard sphere theory

To account for subunit excluded volume while computing K_{c}, we model subunits as effective hard spheres with diameter σ_1. At equilibrium, subunits in the condensate and background must have equal chemical potentials:

$$\mu_{\mathrm{c}} = \mu_{\mathrm{bg}}. \tag{S10}$$

The chemical potentials can be written in terms of ideal and excess (hard sphere) components:

$$\mu_{\mathrm{bg}} = \mu^{\mathrm{ex}}(\rho_1^{\mathrm{bg}}\sigma_1^3) + k_{\mathrm{B}}T\log(\rho_1^{\mathrm{bg}}\sigma_1^3) \tag{S11a}$$

$$\mu_{\mathrm{c}} = \mu^{\mathrm{ex}}(\rho_1^{\mathrm{c}}\sigma_1^3) + k_{\mathrm{B}}T\log(\rho_1^{\mathrm{c}}\sigma_1^3) - \epsilon_{\mathrm{c}}. \tag{S11b}$$

Within the Carnahan-Starling approximation, μ^{ex} is given by [7]:

$$\mu^{\mathrm{ex}}(\rho\sigma_1^3) = \frac{\eta(3\eta^2 - 9\eta + 8)}{(1-\eta)^3}, \quad \eta = \frac{\pi}{6}\rho\sigma_1^3, \tag{S12}$$

where η is the hard sphere packing fraction. Plugging Eqs. S11a, S11b in Eq. S10 and rearranging, we obtain:

$$\frac{\rho_1^{\mathrm{c}}}{\rho_1^{\mathrm{bg}}} = e^{\beta\left[\epsilon_{\mathrm{c}} + \mu^{\mathrm{ex}}(\rho_1^{\mathrm{bg}}\sigma_1^3) - \mu^{\mathrm{ex}}(\rho_1^{\mathrm{c}}\sigma_1^3)\right]}. \tag{S13}$$

Combined with the mass conservation condition

$$\rho_1^{\mathrm{bg}} = (1 + V_{\mathrm{r}})\rho_{\mathrm{T}} - V_{\mathrm{r}}\rho_1^{\mathrm{c}}, \tag{S14}$$

Eq. S13 can be solved self-consistently to obtain ρ_1^{c} and, hence, K_{c}. We set $\sigma_1 = 2.1$ (the 'T' pseudoatom diameter) for the dodecahedron subunits and $\sigma_1 = 3$ (the side length) for the icosahedron subunits. As shown in Fig. 10, we obtain excellent agreement between this hard sphere approximation and results from simulations with $\epsilon_{\mathrm{ss}} = 0$ over a wide range of ϵ_{c}. In contrast, ideal solution theory breaks down for $\epsilon_{\mathrm{c}} \gtrsim 4$ (with $\rho_{\mathrm{T}} = 4 \times 10^{-4}$), corresponding to a packing fraction of $\approx 10\%$.

S3. EQUILIBRIUM ASSEMBLY THEORY

A. Ideal solution theory

Here, we summarize an equilibrium theory for assembly coupled to LLPS in the ideal solution limit (in which excluded volume is neglected). This theory was previously developed in Ref. [8], and a similar theory was presented in Ref. [9]. At equilibrium, intermediates are vanishingly rare [10] and thus we assume that subunits are either monomers or part of complete capsids containing N_{cap} subunits ($N_{\mathrm{cap}} = 12$ for dodecahedral capsids and $N_{\mathrm{cap}} = 20$ for icosahedral capsids). Both monomers and capsids can be located in the condensate or in the background. We denote the concentrations of capsids in the condensate and background as $\rho_{\mathrm{cap}}^{\mathrm{c}}$, $\rho_{\mathrm{cap}}^{\mathrm{bg}}$ respectively. Equilibrium implies that, within each phase (condensate and background), the monomer and (per subunit) capsid chemical potentials are equal, and that monomer and capsid chemical potentials are equal between phases:

$$\mu_1^{\mathrm{bg}} = \mu_{\mathrm{cap}}^{\mathrm{bg}} \tag{S15a}$$

$$\mu_1^{\mathrm{c}} = \mu_{\mathrm{cap}}^{\mathrm{c}} \tag{S15b}$$

$$\mu_1^{\mathrm{bg}} = \mu_1^{\mathrm{c}} \tag{S15c}$$

$$\mu_{\mathrm{cap}}^{\mathrm{bg}} = \mu_{\mathrm{cap}}^{\mathrm{c}}, \tag{S15d}$$

with μ_1^{bg}, μ_1^{c}, $\mu_{\mathrm{cap}}^{\mathrm{bg}}$, and $\mu_{\mathrm{cap}}^{\mathrm{c}}$ as the monomer and (per subunit) capsid chemical potentials in the background and condensate, respectively. Within the ideal solution approximation, the chemical potentials are given by [11]:

$$\mu_1^{\mathrm{bg}} = k_{\mathrm{B}}T\log(\rho_1^{\mathrm{bg}}\sigma_1^3) \tag{S16a}$$

$$\mu_1^{\mathrm{c}} = k_{\mathrm{B}}T\log(\rho_1^{\mathrm{c}}\sigma_1^3) - \epsilon_{\mathrm{c}} \tag{S16b}$$

$$\mu_{\mathrm{cap}}^{\mathrm{bg}} = k_{\mathrm{B}}T\log(\rho_{\mathrm{cap}}^{\mathrm{bg}}\sigma_1^3)/N_{\mathrm{cap}} + f_{\mathrm{capsid}} \tag{S16c}$$

$$\mu_{\mathrm{cap}}^{\mathrm{c}} = k_{\mathrm{B}}T\log(\rho_{\mathrm{cap}}^{\mathrm{c}}\sigma_1^3)/N_{\mathrm{cap}} + f_{\mathrm{capsid}} - \epsilon_{\mathrm{c}}. \tag{S16d}$$

with f_{capsid} as the per-subunit Helmholtz free energy of capsid formation. Additionally, mass conservation yields the constraint:

$$(1 + V_{\mathrm{r}})\rho_{\mathrm{T}} = V_{\mathrm{r}}\rho_1^{\mathrm{c}} + \rho_1^{\mathrm{bg}} + N_{\mathrm{cap}}V_{\mathrm{r}}\rho_{\mathrm{cap}}^{\mathrm{c}} + N_{\mathrm{cap}}\rho_{\mathrm{cap}}^{\mathrm{bg}}. \tag{S17}$$

We solve Eqs. S15 and S17 self-consistently (see Mathematica Notebook 1 available on the Open Science Framework OSFHome: https://osf.io/hq2y8/) to yield the concentrations of monomers and capsids in the background and condensate, and hence the total equilibrium yield:

$$f_{\mathrm{c}}^{\mathrm{equil}} = \frac{N_{\mathrm{cap}}}{\rho_{\mathrm{T}}} \left(\frac{V_{\mathrm{r}}}{1+V_{\mathrm{r}}} \rho_{\mathrm{cap}}^{\mathrm{c}} + \frac{1}{1+V_{\mathrm{r}}} \rho_{\mathrm{cap}}^{\mathrm{bg}} \right). \tag{S18}$$

Following Ref. [8], we note that in the limit of large capsid size ($N \gg 1$), the equilibrium yield can be written as:

$$f_{\mathrm{c}}^{\mathrm{equil}} = \begin{cases} 1 - \frac{\rho_{\mathrm{CAC}}}{\rho_{\mathrm{T}}}, & \rho_{\mathrm{T}} \gg \rho_{\mathrm{CAC}} \\ \left(\frac{\rho_{\mathrm{T}}}{\rho_{\mathrm{CAC}}} \right)^N, & \rho_{\mathrm{T}} \ll \rho_{\mathrm{CAC}}, \end{cases} \tag{S19}$$

with the critical assembly concentration:

$$\rho_{\mathrm{CAC}} = \frac{1 + K_{\mathrm{c}} V_{\mathrm{r}}}{K_{\mathrm{c}}(1+V_{\mathrm{r}})} \left(\frac{1+V_{\mathrm{r}}}{V_{\mathrm{r}}} \right)^{1/N} \rho_{\mathrm{CAC}}^{0}, \tag{S20}$$

and ρ_{CAC}^{0} as the critical assembly concentration without LLPS:

$$\rho_{\mathrm{CAC}}^{0} = N^{-1/N} e^{f_{\mathrm{capsid}}/k_{\mathrm{B}}T}. \tag{S21}$$

Ideal solution theory thus predicts that increasing K_{c} and decreasing V_{r} reduce ρ_{CAC}. For the dodecahedron model, we set $f_{\mathrm{capsid}} = -5k_{\mathrm{B}}T$ for $\epsilon_{\mathrm{ss}} = 6$ (see SI Section S4B for details on this estimate).

B. Hard sphere theory

We now extend ideal solution theory to account for excluded volume. As in the ideal solution theory, at equilibrium we will have equality of chemical potentials between phases and between monomers and capsids within each phase (Eq. S10). To account for excluded volume, we model monomers and capsids as hard spheres with diameters σ_1 and σ_{cap}, respectively. The chemical potentials are now given by:

$$\mu_1^{\mathrm{bg}} = k_{\mathrm{B}}T \log(\rho_1^{\mathrm{bg}} \sigma_1^3) + \mu_1^{\mathrm{ex}}(\rho_1^{\mathrm{bg}}, \rho_{\mathrm{cap}}^{\mathrm{bg}}) \tag{S22a}$$

$$\mu_1^{\mathrm{c}} = k_{\mathrm{B}}T \log(\rho_1^{\mathrm{c}} \sigma_1^3) + \mu_1^{\mathrm{ex}}(\rho_1^{\mathrm{c}}, \rho_{\mathrm{cap}}^{\mathrm{c}}) - \epsilon_{\mathrm{c}} \tag{S22b}$$

$$\mu_{\mathrm{cap}}^{\mathrm{bg}} = k_{\mathrm{B}}T \log(\rho_{\mathrm{cap}}^{\mathrm{bg}} \sigma_1^3)/N_{\mathrm{cap}} + \mu_{\mathrm{cap}}^{\mathrm{ex}}(\rho_1^{\mathrm{bg}}, \rho_{\mathrm{cap}}^{\mathrm{bg}})/N_{\mathrm{cap}} + f_{\mathrm{capsid}} \tag{S22c}$$

$$\mu_{\mathrm{cap}}^{\mathrm{c}} = k_{\mathrm{B}}T \log(\rho_{\mathrm{cap}}^{\mathrm{c}} \sigma_1^3)/N_{\mathrm{cap}} + \mu_{\mathrm{cap}}^{\mathrm{ex}}(\rho_1^{\mathrm{c}}, \rho_{\mathrm{cap}}^{\mathrm{c}})/N_{\mathrm{cap}} + f_{\mathrm{capsid}} - \epsilon_{\mathrm{c}}. \tag{S22d}$$

Here, μ_1^{ex} and $\mu_{\mathrm{cap}}^{\mathrm{ex}}$ are excess chemical potentials for a binary mixture of hard spheres [12, 13]:

$$\begin{aligned} \mu_\nu^{\mathrm{ex}}(\rho_1, \rho_{\mathrm{cap}}) = {} & c_0(\eta) + c_1(\eta) \frac{M_1 M_2}{M_3} \frac{\sigma_1}{M_1} + \left(c_1(\eta) \frac{M_1 M_2}{M_3} + 3a_2(\eta) \frac{M_2^3}{M_3^2} \right) \frac{\sigma_1^2}{M_2} \\ & + \left[\eta c_0'(\eta) + (\eta c_1'(\eta) - c_1(\eta)) \frac{M_1 M_2}{M_3} + (\eta a_2'(\eta) - 2a_2(\eta)) \frac{M_2^3}{M_3^2} \right] \frac{\sigma_1^3}{M_3} \end{aligned} \tag{S23a}$$

$$\eta = \frac{\pi}{6}(\rho_1 + \rho_{\mathrm{cap}}) M_3 \tag{S23b}$$

$$c_0(\eta) = -\log(1-\eta) \tag{S23c}$$

$$c_1(\eta) = \frac{3\eta}{1-\eta} \tag{S23d}$$

$$a_2(\eta) = \log(1-\eta) + \frac{\eta}{(1-\eta)^2} \tag{S23e}$$

$$M_i = \frac{\sum_\nu \rho_\nu \sigma_\nu^i}{\sum_\nu \rho_\nu}, \tag{S23f}$$

where $\nu = 1, \mathrm{cap}$ and the prime symbol denotes a derivative with respect to η.

We solve Eqs. S15 and S17 using the chemical potentials in Eq. S22 (see Mathematica Notebook 2 available on the Open Science Framework OSFHome: https://osf.io/hq2y8/) to obtain the equilibrium concentrations of monomers

and capsids in each phase, and hence the equilibrium yield. For the dodecahedron model, we set $\sigma_1 = 2.1$ (the 'T' pseudoatom diameter), $\sigma_{\rm cap} = 5.1$, and $f_{\rm capsid} = -5k_{\rm B}T$ (see SI Section S4A for details on estimating $\sigma_{\rm cap}$). We plot both the ideal solution and hard sphere predictions for $f_{\rm c}^{\rm equil}$ versus $\rho_{\rm T}$ for different values of $\epsilon_{\rm c}$ in Fig. S1. For $\epsilon_{\rm c} = 0$ the ideal solution and hard sphere results closely match, as expected for a dilute solution. On the other hand, when a condensate is present ($\epsilon_{\rm c} > 0$), the hard sphere predictions deviate significantly from ideal solution theory beyond a threshold concentration, reflecting the high concentration of subunits within the condensate.

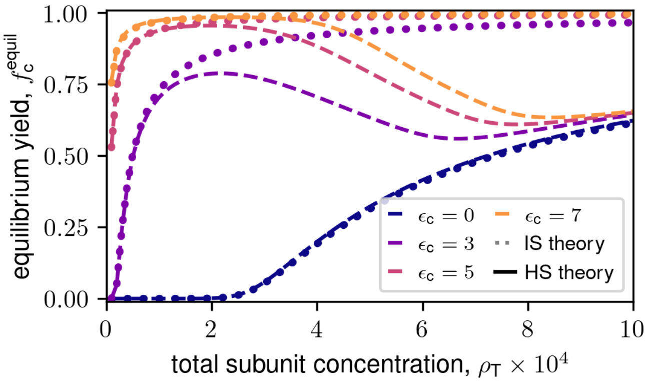

FIG. S1. Equilibrium yield versus total subunit concentration, predicted by the HS (solid lines) and IS (dotted lines) theories for indicated values of $\epsilon_{\rm c}$. Parameters are: the subunit binding well depth $\epsilon_{\rm ss} = 6$, and condensate volume fraction $V_{\rm r} = 5.0 \times 10^{-3}$, and final simulation time $t_{\rm F} = 6 \times 10^5$.

S4. ESTIMATING PARAMETERS FOR THE THEORETICAL MODEL

A. Estimating $\sigma_{\rm cap}$

To estimate the capsid effective hard sphere diameter, we computed the capsid radial distribution function $g(r)$ (Fig. S2) at a parameter set where condensates are densely packed with capsids ($\rho_{\rm T} = 10^{-3}$, $V_{\rm r} = 5.0 \times 10^{-3}$, $\epsilon_{\rm ss} = 6$, $t_{\rm F} = 6 \times 10^5$). We took $\sigma_{\rm cap}$ to be halfway between the location of the first peak of $g(r)$ (≈ 5.3) and the smallest value of r for which $g(r)$ is nonzero (≈ 4.9), giving an estimate of $\sigma_{\rm cap} \approx 5.1$. We also estimated $\sigma_{\rm cap}$ via an alternative route, in which we considered assembled capsids as rigid bodies and computed the minimum distance that two capsids could approach each other. In snapshots of densely packed capsids, we often observed capsids touching either face-to-face or vertex-to-face (with a 'T' pseudoatom on one capsid nestled between a face consisting of three 'T' pseudoatoms on another capsid). We therefore computed the distance of closest approach in either of these two orientations, obtaining values of ≈ 4.8 for face-to-face and ≈ 5.3 for vertex-to-face (see Mathematica Notebook 3 available on the Open Science Framework OSFHome: https://osf.io/hq2y8/). The average of these two values is approximately equal to our previous estimate of $\sigma_{\rm cap} \approx 5.1$.

B. Estimating $f_{\rm capsid}$

To estimate $f_{\rm capsid}$, we used previously reported approximate free energies for capsid intermediates up to, but not including, a full capsid in the dodecahedron model (see Ref. [14]). We plot these free energies for $\epsilon_{\rm ss} = 6$ in Fig. S3. For intermediate sizes $n \gtrsim 3$, the free energy to add an additional subunit is roughly independent of n, $\approx -5.5k_{\rm B}T$, until the 11th subunit binds. The accuracy of these free energy estimates is limited by sampling for larger intermediates and we would expect a more nonlinear dependence on n (since more bonds are formed on average as intermediates grow and the binding entropy is expected to be sub-linear in number of bonds [15, 16]). Furthermore, because the insertion of the 12th subunit (making a complete capsid) makes more bonds (5) compared to previous subunits than any other, this reaction is irreversible on simulation timescales. That is, we never see a complete capsid disassemble at binding energy values for which assembly occurs. Given the irreversibility and larger number of bonds formed, we anticipate that the free energy of adding the final subunit is significantly higher than that of adding previous subunits. We thus roughly approximate the free energy of adding the final subunit as $-11k_{\rm B}T$, roughly twice that of adding previous subunits, such that the total capsid free energy is $\approx -60k_{\rm B}T$. Thus, the free energy per subunit

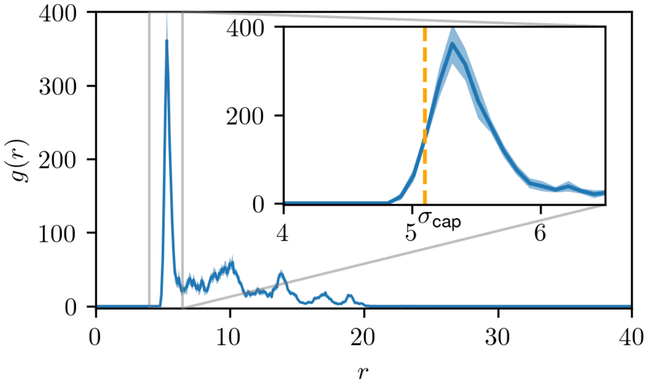

FIG. S2. Capsid radial distribution function $g(r)$ versus center-of-mass distance r, computed from simulations with $V_{\mathrm{r}} = 5.0 \times 10^{-3}$, $\rho_{\mathrm{T}} = 10^{-3}$, $\epsilon_{\mathrm{ss}} = 6$, and $\epsilon_{\mathrm{c}} = 7$. Here $g(r)$ was computed by averaging over the final configurations of 10 independent assembly trajectories, with error bars representing the standard error. The inset shows a zoomed-in view of the first peak, and the orange dashed line shows the capsid effective hard sphere diameter σ_{cap}.

is $f_{\text{capsid}} \approx -5k_{\mathrm{B}}T$. However, the capsid free energy will depend on ϵ_{ss}. To estimate this dependence, we use the previously-reported [17] dependence of dimerization free energy on ϵ_{ss}, $\sim -1.56\epsilon_{\mathrm{ss}} + Ts$, where s is the per-subunit entropy. Assuming that the capsid free energy depends on ϵ_{ss} in the same way as the dimerization free energy, we thus write:

$$f_{\text{capsid}}(\epsilon_{\mathrm{ss}}) = -1.56\epsilon_{\mathrm{ss}} + Ts. \tag{S24}$$

We then use the fact that $f_{\text{capsid}}(6k_{\mathrm{B}}T) = -5k_{\mathrm{B}}T$ to obtain $Ts = 4.36k_{\mathrm{B}}T$.

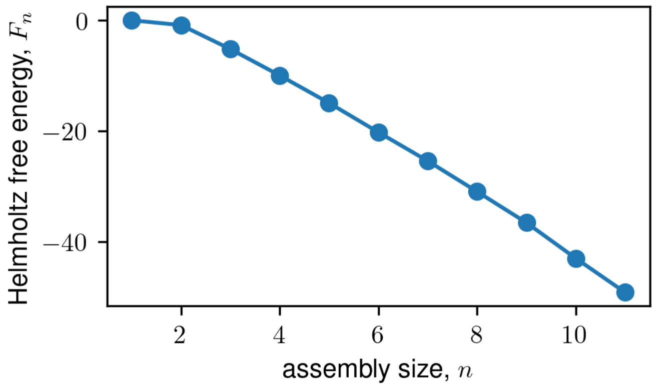

FIG. S3. Helmholtz free energy F_n as a function of assembly size n. Data taken from Ref. [17].

S5. YIELD VS ϵ_{ss} AT DIFFERENT TIMES

Fig. S4a,b plots the yields from Fig. 6 at different times for $\epsilon_{\mathrm{c}} = 7$ and $\epsilon_{\mathrm{c}} = 0$ respectively. With LLPS, capsid production is orders of magnitude faster and yields approach much closer to the estimated equilibrium value within the finite simulation timescale. Moreover, assembly rates are increased even in conditions leading to malformed assemblies ($\epsilon_{\mathrm{ss}} \geq 7.5$). However, consistent with the results described in the main text, assembly growth slows at later times. While the slowdown with LLPS primarily occurs due to the excluded volume effects described above, assembly rates in bulk solution decrease as monomers are depleted, leading to a growth in the size of the critical nucleus size and the associated free energy barrier [18].

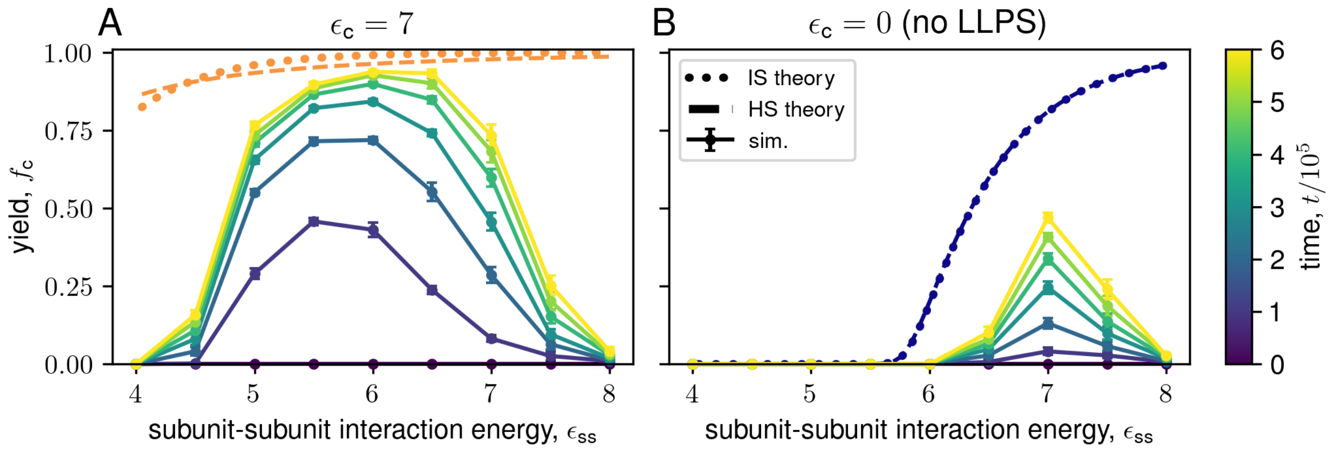

FIG. S4. Yield as a function of ϵ_{ss} at indicated times for (A) $\epsilon_c = 7$ and (B) $\epsilon_c = 0$ (i.e. without LLPS), for the same parameters as Fig. 6A. The equilibrium hard sphere and ideal solution theories are shown as dashed and dotted lines respectively.

S6. SCALING THEORY FOR ASSEMBLY KINETICS

The kinetics of LLPS-mediated assembly involves diffusion of subunits into the condensate, formation of critical nuclei, and elongation (growth) of nuclei into complete capsids. Previous work [8] predicted how LLPS affects the maximum assembly rate and median assembly timescale by noting that nucleation is typically the rate-limiting step in self-assembly, and by assuming that the primary effect of LLPS is to increase the nucleation rate by increasing the local subunit concentration. Also assuming that the critical nucleus size n_{nuc} is independent of conditions, the maximum assembly rate r_{max} and median assembly time $\tau_{1/2}$ were predicted to depend on the partition coefficient K_c and condensate volume fraction V_r as:

$$\begin{aligned} r_{\text{max}}(V_r, K_c) \approx& r_{\text{nuc}}(V_r, K_c) = s_{\text{nuc}} r^0_{\text{nuc}} \\ \tau_{1/2}(V_r, K_c) =& \tau^0_{1/2}/s_{\text{nuc}} \\ s_{\text{nuc}} =& \left(\frac{1+V_r}{1+K_c V_r}\right)^{n_{\text{nuc}}} \frac{1+V_r K_c^{n_{\text{nuc}}}}{1+V_r} \\ \approx& V_r / \left(V_r + 1/K_c\right)^{n_{\text{nuc}}}. \end{aligned} \tag{S25}$$

Here, r^0_{nuc} and $\tau^0_{1/2}$ are the initial nucleation rate (before significant subunit depletion has occurred) and median assembly time in the absence of LLPS ($\epsilon_c = 0$). The approximate expression for the nucleation speedup s_{nuc} is valid for $K_c^{n_{\text{nuc}}} \gg 1/V_r$, such that nucleation occurs only in the compartment.

This previously-developed scaling theory assumes diffusion is very fast compared to nucleation. Yet this assumption is violated in our simulations, particularly for high ϵ_c, as shown in Fig. 7C,F in the main text. We therefore extend the scaling theory to account for diffusive timescales. First, we note that the maximum assembly rate will be limited by the diffusive flux of subunits into the condensate, (assuming that assembly occurs exclusively within the condensate) with a "forward" rate (i.e. diffusion *into* the condensate) given by: $4\pi R_c D \rho_1^{\text{bg}}$ ($\approx 4\pi R_c D \rho_T$, assuming minimal subunit depletion). Second, we note that the median assembly time must account for the characteristic timescale for subunit diffusion between condensate and background (not just the forward flux of subunits into the compartment). To obtain this timescale, we follow the Supporting Material of Ref. [8] and write the dynamics of subunit partitioning between the background and compartment as:

$$\frac{d\rho_1^c}{dt} = \frac{4\pi R_c D}{V_c}\left(\rho_1^{\text{bg}} - \rho_1^c/K_c\right) \tag{S26a}$$

$$\frac{d\rho_1^{\text{bg}}}{dt} = -\frac{V_c}{V_{\text{bg}}}\frac{d\rho_1^c}{dt}. \tag{S26b}$$

Assuming initial concentrations ρ_T in both the background and condensate results in:

$$\rho_1^c(t) = \left(\frac{1+V_r}{V_r+1/K_c}\right)\rho_T + \left(1 - \frac{1+V_r}{V_r+1/K_c}\right)\rho_T e^{-(4\pi R_c D(V_r+K_c^{-1})/V_c)t} \tag{S27}$$

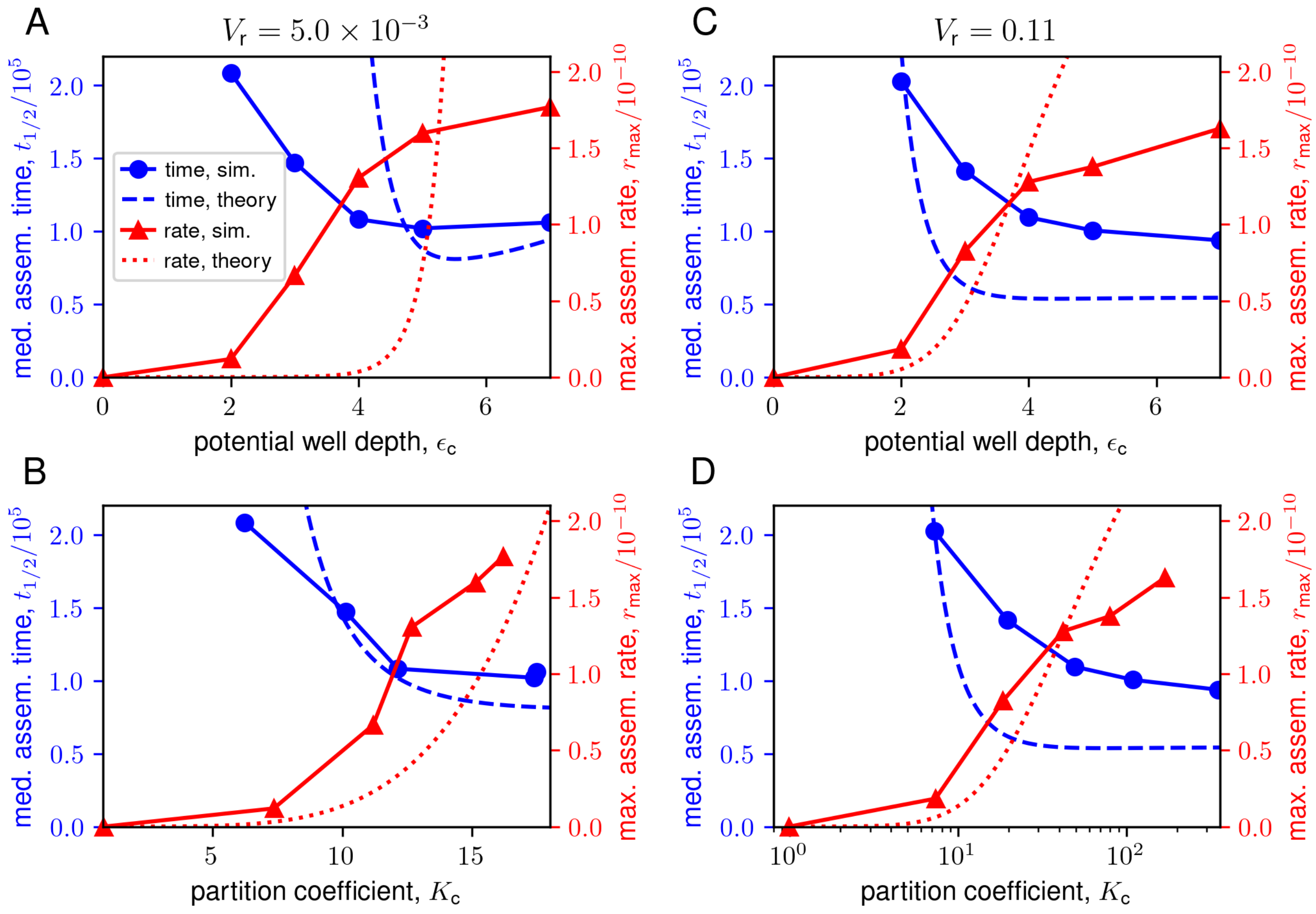

FIG. S5. Comparison of simulations and scaling theory for median assembly time $\tau_{1/2}$ and maximum assembly rate $r_{\max}$. Panels A,B show results for $V_r = 5.0 \times 10^{-3}$, and panels C,D show results for $V_r = 0.11$. Panels A,C show results as a function of potential well depth ϵ_c, while panels B,D show the same results as a function of partition coefficient K_c. The scaling theory curves (Eq. S25) were fit by eye to the simulation data for $V_r = 0.11$ as a function of ϵ_c, resulting in fitting parameters $r^0_{\rm nuc} = 10^{-9}$, $\tau^0_{1/2} = 3 \times 10^7$, and $n_{\rm nuc} = 5$.

We identify $V_c/\left[(V_r + K_c^{-1})4\pi R_c D\right]$ as a diffusive timescale. However, this expression fails to account for the subunits that will form capsids. To roughly capture the diffusion timescale associated with those subunits, we add a term $f_c V/(4\pi R_c D)$, resulting in a total diffusive timescale:

$$\tau_D \approx \frac{V_c/(V_r + 1/K_c) + f_c V}{4\pi R_c D}, \tag{S28}$$

which is Eq. 4 in the main text. Thus, our new scaling estimates for $r_{\max}$ and $\tau_{1/2}$ are:

$$r_{\max}(V_r, K_c) = \frac{s_{\rm nuc} r^0_{\rm nuc} 4\pi R_c D \rho_T}{s_{\rm nuc} r^0_{\rm nuc} + 4\pi R_c D \rho_T} \frac{\rho_T}{N_{\rm cap}} \tag{S29a}$$

$$\tau_{1/2}(V_r, K_c) = \tau^0_{1/2}/s_{\rm nuc} + \tau_D/2. \tag{S29b}$$

(We have included the factor of $\rho_T/N_{\rm cap}$ to give $r_{\max}$ units of capsid concentration per unit time.)

We compare the theoretical predictions of Eq. S29 to simulation results in Fig. S5. Because we observe no assembly without LLPS for the parameters used in Fig. 7, we treat $r^0_{\rm nuc}$ and $\tau^0_{1/2}$, as well as $n_{\rm nuc}$, as fitting parameters. We choose (by eye) $r^0_{\rm nuc} = 10^{-9}$, $\tau^0_{1/2} = 3 \times 10^7$, and $n_{\rm nuc} = 5$ to yield rough agreement with the simulation results for $V_r = 0.11$, and then use the same parameter values for $V_r = 5 \times 10^{-3}$. We find that the diffusive flux term $4\pi R_c D \rho_T$ has little effect on $r_{\max}$ because here $4\pi R_c D \rho_T \gg s_{\rm nuc} r^0_{\rm nuc}$; however, we note that the true diffusive flux

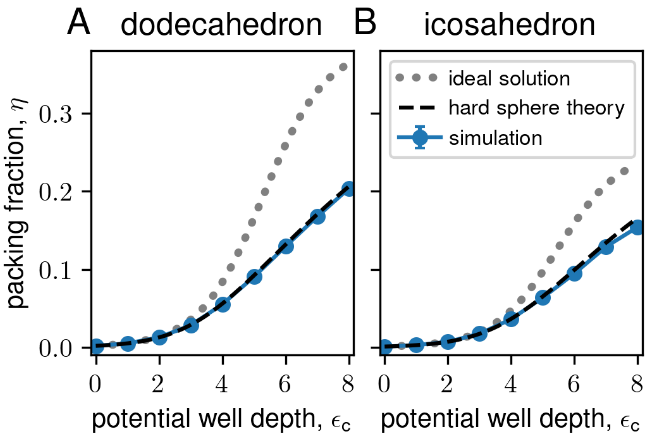

FIG. S6. Packing fraction in the condensate $\eta = \frac{4}{3}\pi(\sigma_1/2)^3\rho_1^c$ as a function of potential well-depth ϵ_c for the (A) dodecahedron model and (B) icosahedron model. Error bars are smaller than the size of the plotted points.

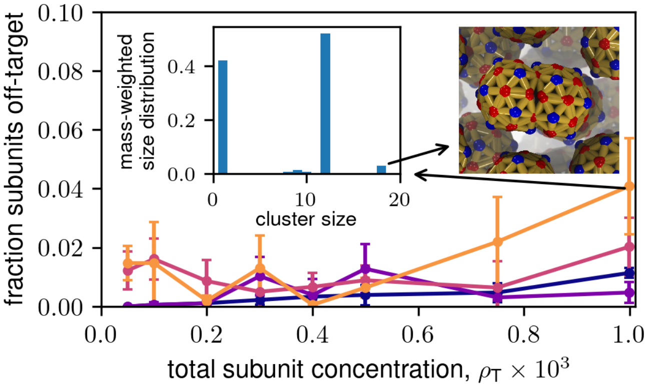

FIG. S7. Fraction of subunits in off-target structures (neither monomers nor capsids) versus total subunit concentration for different partition coefficients. The inset plot shows the mass-weighted cluster size distribution for $\rho_T = 10^{-3}$, $\epsilon_c = -7k_BT$. The two largest peaks at cluster sizes of 1 and 12 correspond to monomers and capsids, respectively. The next largest peak is at 18, corresponding to partial capsids bound together as shown in the snapshot.

could be significantly smaller due to the excluded volume of subunits and capsids in the condensate "blocking" entry of additional subunits. However, including $\tau_D/2$ in $\tau_{1/2}$ correctly captures the saturation of $\tau_{1/2}$ with ϵ_c (without τ_D, the theory severely underestimates the median assembly time at high ϵ_c).

When we assume the ideal solution partition coefficient ($K_c^{IS} = e^{\beta\epsilon_c}$) and plot the scaling prediction as a function of ϵ_c (Fig. S5A,C), we observe poor agreement, which worsens with increasing ϵ_c, particularly for r_{max}. However, we observe much better agreement when we instead use the values of K_c^{meas} measured at the time of maximum assembly rate for r_{max} and at the median assembly time for $\tau_{1/2}$ (Fig. S5B,D). The agreement particularly improves for $V_r = 5 \times 10^{-3}$, where the K_c^{meas} values deviate significantly from K_c^{IS}.

S7. ADDITIONAL SUPPLEMENTARY FIGURES

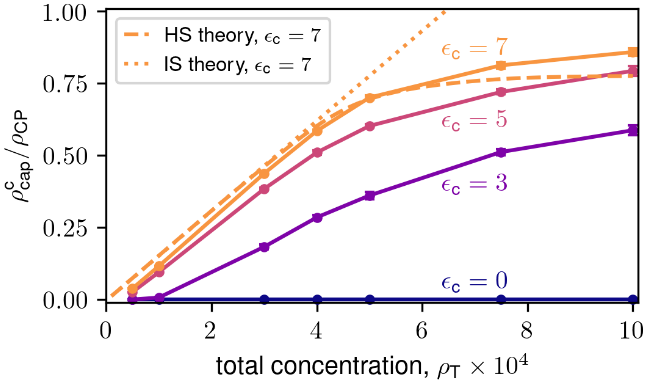

FIG. S8. Concentration of capsids in the condensate normalized by the estimated close-packing density, $\rho^{c}_{\text{cap}}/\rho_{\text{CP}}$, versus total subunit concentration for the same parameters as in Fig. 3.

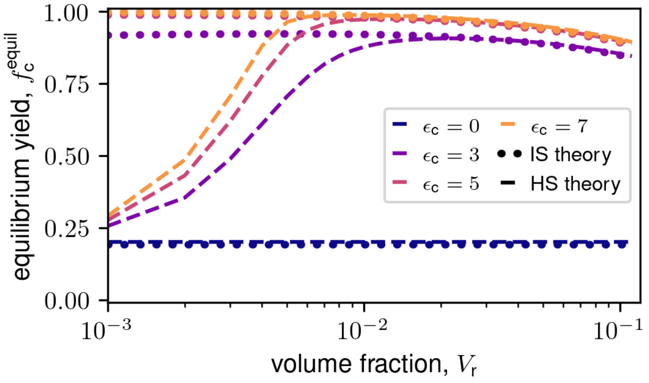

FIG. S9. Equilibrium yield versus volume fraction for indicated partition coefficient parameters, shown for the IS (dots) and HS (solid) theories. Parameters are $\epsilon_{\text{ss}} = 6$, $\rho_{\text{T}} = 4.00 \times 10^{-4}$, and $t_{\text{F}} = 6 \times 10^{5}$.

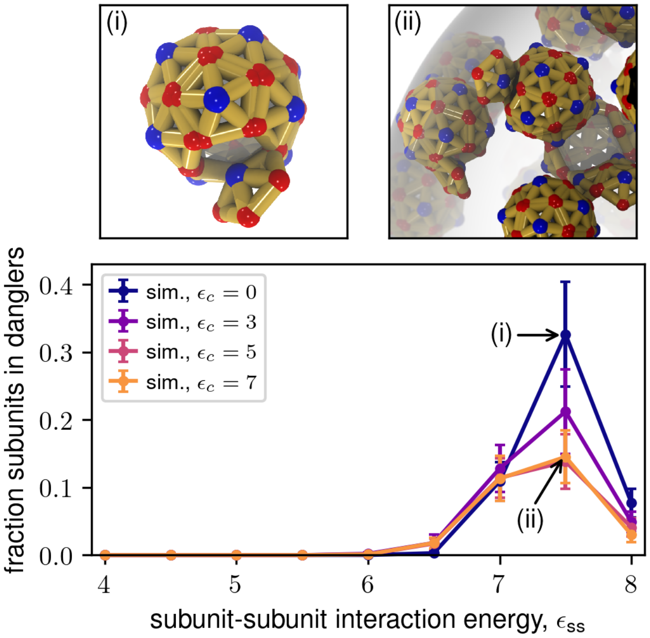

FIG. S10. Fraction of subunits in danglers versus subunit-subunit interaction energy. Snapshots above plot correspond to labeled points: (i) dangler in bulk solution with $\epsilon_c = 0$ (ii) danglers in a condensate with $\epsilon_c = 7$. Parameters are: $\rho_T = 4 \times 10^{-4}$, $V_r = 5.0 \times 10^{-3}$, and $t_F = 10^6$

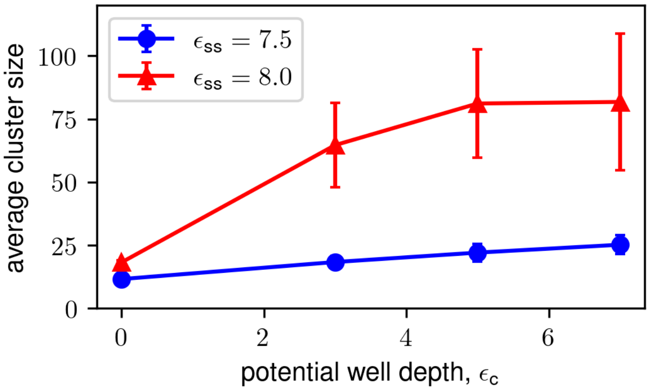

FIG. S11. The average cluster size at long times is shown as a function of ϵ_c for $\epsilon_{ss} = 7.5, 8$. Parameters are: $\rho_T = 4 \times 10^{-4}$, $V_r = 5.0 \times 10^{-3}$, $t_F = 6 \times 10^5$.

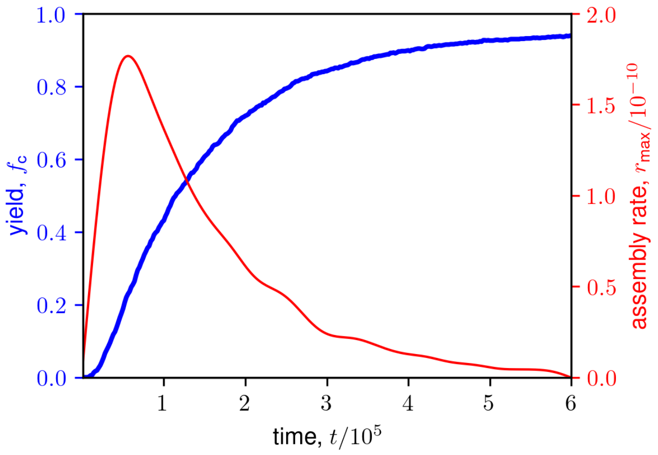

FIG. S12. Example of assembly rate calculation from yield vs. time data. The yield is the thick blue line; the assembly rate is the thin red line. The assembly rate is computed by convolving the yield curve with the derivative of a Gaussian; we set the smoothing parameter (i.e. the standard deviation of the Gaussian) to $\sigma = 10$. Simulation parameters are: $\epsilon_c = 7$, $\epsilon_{ss} = 6$, $V_r = 5.0 \times 10^{-3}$, $\rho_T = 4 \times 10^{-4}$.

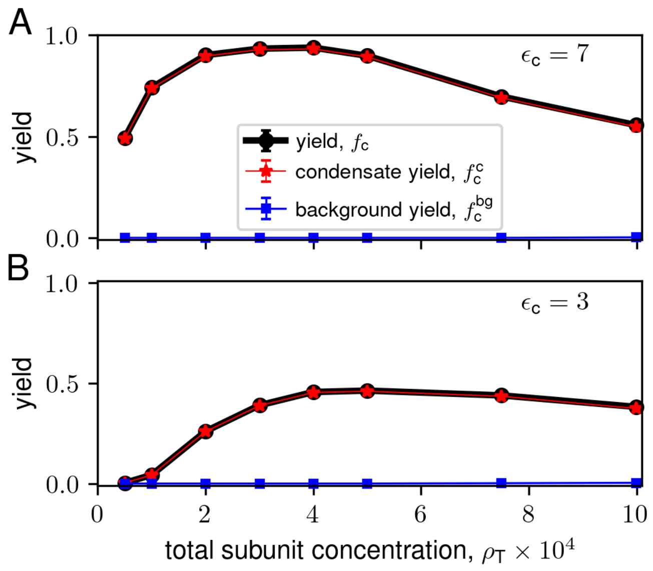

FIG. S13. Total (f_c), condensate (f_c^c), and background (f_c^{bg}) yields as a function of total subunit concentration ρ_T for simulations with (A) $\epsilon_c = 7$ and (B) $\epsilon_c = 3$. The yield is almost entirely due to capsids in the condensate, even for modest partitioning ($\epsilon_c = 3$). Other parameters are: $V_r = 5.0 \times 10^{-3}$, and $t_F = 6 \times 10^5$.

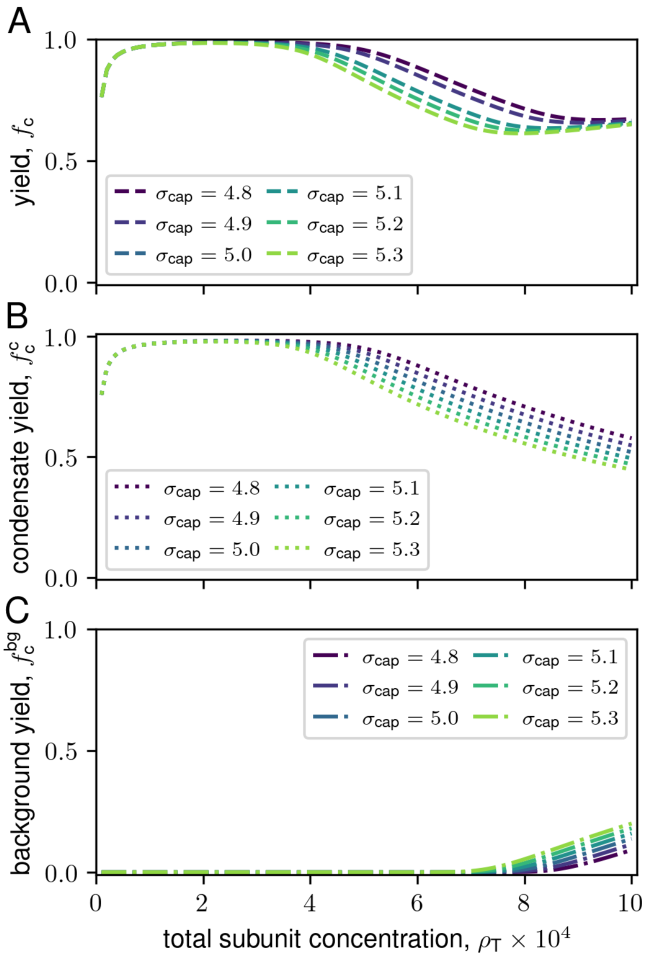

FIG. S14. Yield (A), condensate yield (B), and background yield (C) versus total subunit concentration ρ_T for different capsid diameters σ_{cap} within the equilibrium hard sphere theory. Reducing the capsid excluded volume (lower σ_{cap}) increases the total and condensate yields over a range of concentrations, while reducing the background yield at high concentrations. Parameters are: $\epsilon_c = 7$, $V_r = 5.0 \times 10^{-3}$.

S8. MOVIE DESCRIPTIONS

- **Movie S1**: Trajectory showing no assembly without LLPS. Parameters are: $\epsilon_{\mathrm{c}} = 0$, $\epsilon_{\mathrm{ss}} = 6$, $\rho_{\mathrm{T}} = 4 \times 10^{-4}$.
- **Movie S2**: Capsid assembly trajectory without LLPS. Parameters are: $\epsilon_{\mathrm{c}} = 0$, $\epsilon_{\mathrm{ss}} = 7$, $\rho_{\mathrm{T}} = 4 \times 10^{-4}$.
- **Movie S3**: Capsid assembly trajectory with LLPS for relatively weak subunit partitioning. Parameters are: $\epsilon_{\mathrm{c}} = 3$, $\epsilon_{\mathrm{ss}} = 6$, $\rho_{\mathrm{T}} = 4 \times 10^{-4}$, $V_{\mathrm{r}} = 5.0 \times 10^{-3}$.
- **Movie S4**: Capsid assembly trajectory with LLPS for relatively stong subunit partitioning. Parameters are: $\epsilon_{\mathrm{c}} = 7$, $\epsilon_{\mathrm{ss}} = 6$, $\rho_{\mathrm{T}} = 4 \times 10^{-4}$, $V_{\mathrm{r}} = 5.0 \times 10^{-3}$.
- **Movie S5**: Capsid assembly trajectory with LLPS for a relatively large condensate volume fraction. Parameters are: $\epsilon_{\mathrm{c}} = 7$, $\epsilon_{\mathrm{ss}} = 6$, $\rho_{\mathrm{T}} = 4 \times 10^{-4}$, $V_{\mathrm{r}} = 0.11$.
- **Movie S6**: Capsid assembly trajectory without LLPS at high subunit binding affinity, exhibiting malformed structures. Parameters are: $\epsilon_{\mathrm{c}} = 0$, $\epsilon_{\mathrm{ss}} = 8$, $\rho_{\mathrm{T}} = 4 \times 10^{-4}$.
- **Movie S7**: Capsid assembly trajectory with LLPS at high subunit binding affinity, exhibiting malformed structures. Parameters are: $\epsilon_{\mathrm{c}} = 7$, $\epsilon_{\mathrm{ss}} = 8$, $\rho_{\mathrm{T}} = 4 \times 10^{-4}$, $V_{\mathrm{r}} = 5.0 \times 10^{-3}$.

---

[1] C. Sigl, E. M. Willner, W. Engelen, J. A. Kretzmann, K. Sachenbacher, A. Liedl, F. Kolbe, F. Wilsch, S. A. Aghvami, U. Protzer, M. F. Hagan, S. Fraden, and H. Dietz, Programmable icosahedral shell system for virus trapping, Nature Materials **20**, 1281 (2021).

[2] W.-S. Wei, A. Trubiano, C. Sigl, S. Paquay, H. Dietz, M. F. Hagan, and S. Fraden, Hierarchical assembly is more robust than egalitarian assembly in synthetic capsids, Proceedings of the National Academy of Sciences **121**, e2312775121 (2024).

[3] J. D. Weeks, D. Chandler, and H. C. Andersen, Role of Repulsive Forces in Determining the Equilibrium Structure of Simple Liquids, The Journal of Chemical Physics **54**, 5237 (1971).

[4] J. A. Anderson, J. Glaser, and S. C. Glotzer, HOOMD-blue: A Python package for high-performance molecular dynamics and hard particle Monte Carlo simulations, Computational Materials Science **173**, 109363 (2020).

[5] M. P. Allen and D. J. Tildesley, *Computer Simulation of Liquids* (Clarendon Press ; Oxford University Press, Oxford England New York, 1989).

[6] H. Kamberaj, R. J. Low, and M. P. Neal, Time reversible and symplectic integrators for molecular dynamics simulations of rigid molecules, The Journal of Chemical Physics **122**, 224114 (2005).

[7] P. Attard, Simulation of the chemical potential and the cavity free energy of dense hard-sphere fluids, The Journal of Chemical Physics **98**, 2225 (1993).

[8] M. F. Hagan and F. Mohajerani, Self-assembly coupled to liquid-liquid phase separation, PLOS Computational Biology **19**, e1010652 (2023).

[9] G. Bartolucci, I. S. Haugerud, T. C. T. Michaels, and C. A. Weber, The interplay between biomolecular assembly and phase separation, eLife **13**, 10.7554/eLife.93003.1 (2024).

[10] M. F. Hagan and G. M. Grason, Equilibrium mechanisms of self-limiting assembly, Reviews of Modern Physics **93**, 025008 (2021).

[11] J. N. Israelachvili, D. John Mitchell, and B. W. Ninham, Theory of self-assembly of hydrocarbon amphiphiles into micelles and bilayers, Journal of the Chemical Society, Faraday Transactions 2: Molecular and Chemical Physics **72**, 1525 (1976).

[12] G. A. Mansoori, N. F. Carnahan, K. E. Starling, and T. W. Leland, Equilibrium Thermodynamic Properties of the Mixture of Hard Spheres, The Journal of Chemical Physics **54**, 1523 (1971).

[13] D. M. Heyes and A. Santos, Chemical potential of a test hard sphere of variable size in a hard-sphere fluid, The Journal of Chemical Physics **145**, 214504 (2016).

[14] A. Trubiano, Self-Assembly Analysis Suite for HOOMD, https://github.com/onehalfatsquared/SAASH (2024).

[15] M. F. Hagan and D. Chandler, Dynamic pathways for viral capsid assembly, Biophysical Journal **91**, 42 (2006).

[16] M. F. Hagan, O. M. Elrad, and R. L. Jack, Mechanisms of kinetic trapping in self-assembly and phase transformation, Journal of Chemical Physics **135**, 104115 (2011).

[17] A. Trubiano and M. F. Hagan, Markov state model approach to simulate self-assembly, Phys. Rev. X **14**, 041063 (2024).

[18] M. F. Hagan and O. Elrad, Understanding the concentration dependence of viral capsid assembly kinetics - the origin of the lag time and identifying the critical nucleus size, Biophysical Journal **98**, 1065 (2010).
